# Synthesis and Evaluation of New Quinoxaline Derivatives of Dehydroabietic Acid as Potential Antitumor Agents

**DOI:** 10.3390/molecules22071154

**Published:** 2017-07-11

**Authors:** Wen Gu, Shuang Wang, Xiaoyan Jin, Yaliang Zhang, Dawei Hua, Tingting Miao, Xubing Tao, Shifa Wang

**Affiliations:** 1 Jiangsu Key Lab of Biomass-Based Green Fuels and Chemicals, College of Chemical Engineering, Nanjing Forestry University, Nanjing 210037, China; loniuniu@126.com (S.W.); JinXYsweet@163.com (X.J.); davidhua22@gmail.com (D.H.); 13775445886@163.com (T.M.); taoxubing0126@163.com (X.T.); wangshifa65@163.com (S.W.); 2 The State Key Lab of Pharmaceutical Biotechnology, School of Life Sciences, Nanjing University, Nanjing 210093, China; zhangyaliang@smail.nju.edu.cn

**Keywords:** dehydroabietic acid, quinoxaline, antitumor activity, cell cycle, apoptosis

## Abstract

A series of new quinoxaline derivatives of dehydroabietic acid (DAA) were designed and synthesized as potential antitumor agents. Their structures were characterized by IR, ^1^H-NMR, ^13^C-NMR, and MS spectra and elemental analyses. All the new compounds were screened for their in vitro antiproliferative activities against three human cancer cell lines (MCF-7, SMMC-7721 and HeLa) and noncancerous human hepatocyte cells (LO2). A cytotoxic assay manifested that compound **4b** showed the most potent cytotoxic activity against the three cancer cell lines, with IC_50_ values of 1.78 ± 0.36, 0.72 ± 0.09 and 1.08 ± 0.12 μM, respectively, and a substantially lower cytotoxicity to LO2 cells (IC_50_: 11.09 ± 0.57 μM). Moreover, the cell cycle analysis suggested that compound **4b** caused cell cycle arrest of SMMC-7721 cells at the G0/G1 phase. In a Hoechst 33258 staining assay, compound **4b** caused considerable morphological changes of the nuclei of SMMC-7721 cells, correlated with cell apoptosis. In addition, an Annexin V-FITC/PI dual staining assay confirmed that compound **4b** could induce the apoptosis of SMMC-7721 cells in a dose-dependent manner.

## 1. Introduction

Cancer, as one of the most serious clinical problems, is still threatening people’s health throughout the world [1]. Despite the crucial role of cancer chemotherapy, the lack of antitumor selectivity has become one main barrier in the development of effective anticancer drugs. The general toxicity substantially limits the clinical development of some anticancer agents with significant preclinical efficacy [2]. Therefore, there is continuous need for chemists to develop novel anticancer agents with higher efficacy and lower side effects. With diverse structures, natural products have proved to be a rich source for anticancer drugs such as paclitaxel, vinblastine, doxorubicin etc., and a number of their derivatives have been developed and used clinically in recent years [3,4]. Statistically, at least 60% of anticancer agents originate from natural compounds [5], which suggest that the derivation of bioactive natural products is a promising strategy for the discovery of new anticancer drugs.

Dehydroabietic acid (DAA, **1**) is a naturally occurring diterpene resin acid abundant in *Pinus* rosin or commercial disproportionated rosin. Recent reports indicate that DAA and its derivatives exhibited a wide range of biological activities, such as antibacterial [6], antifungal [7], anti-inflammatory [8], antiulcer [9], antiviral [10], antiherpetic, antidengue [11] and anti-aging [12] activities. In addition, a number of DAA derivatives have been reported in recent years to possess significant antitumor properties through DNA binding [13], apoptosis [14] or oncosis inducing [15] mechanisms. These findings suggest that DAA is a promising starting material for the discovery of new anticancer agents.

Heterocycles play a prominent role in the biological activity of many natural products [16,17]. Incorporating heteroatoms into a molecular scaffold increases the drug-like properties of molecules, providing solubility, hydrogen bonding and rigidity. The presence of lone-electron pairs on heteroatoms benefits the formation of hydrogen bonding with water, which increases the solubility, and likely improves the binding of the molecule to its potential targets [18]. Quinoxalines have shown a variety of biological activities, including antibacterial, antiviral, herbicidal, anti-inflammatory and antitumor activities [19,20,21,22,23], which indicates that they are an important class of *N*-containing heterocycles in organic synthesis and drug discovery [24]. Some drug candidates bearing quinoxaline core structures are currently under clinical trials for anticancer therapeutic purposes [25]. Compounds containing azole heterocycles, for example, imidazole, triazole, tetrazole, etc., have also been reported as potential anticancer agents because these motifs are ready to interact with particular receptors or enzyme active sites [26,27]. In addition, previous literature indicates that the introduction of a flexible alkylamine side chain into some heterocyclic compounds may also improve their antitumor potencies [28]. In view of these findings, a series of novel 2,3-disubstituted quinoxaline derivatives of DAA containing alkylamine or azole moieties were designed and synthesized. The antiproliferative activities and apoptosis-inducing properties of the target compounds are also presented herein.

## 2. Results and Discussion

### 2.1. Chemistry

**Scheme 1:**
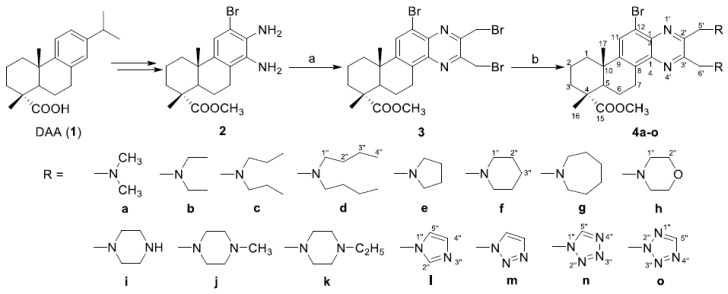
Synthetic route for the target compounds (**4a**–**o**) from dehydroabietic acid (DAA, **1**). Reagents and conditions: (**a**) 1,4-dibromo-2,3-butanedione, HOAc, 120 °C, 2 h; (**b**) corresponding amine or heterocycle, K_2_CO_3_, KI, MeCN, 85 °C, 8–12 h.

The synthetic route for the target compounds (**4a**–**o**) is shown in Scheme 1. Briefly, the diamino intermediate **2** was synthesized from DAA (**1**) based on the method previously reported [29]. Then, compound **2** was reacted with 1,4-dibromo-2,3-butanedione to give the key 2,3-bis(bromomethyl)-quinoxaline derivative (**3**) in 78% yield. Subsequently, compound **3** was converted to the target quinoxaline derivatives containing different *N*-containing side chains (**4a**–**o**) in 32–63% yield, by reacting with different amines or azoles in the presence of K_2_CO_3_ and KI. All the synthesized compounds were purified by recrystallization or silica gel column chromatography with a gradient elution of petroleum ether–acetone or CH_2_Cl_2_–MeOH, and their structures were characterized through IR, ^1^H-NMR, ^13^C-NMR and ESIMS spectra and elemental analyses. In a typical example, the ESIMS of compound **4h** displayed two peaks at *m*/*z* 601 and 603, corresponding to the quasimolecular ions [M + H]^+^, which suggested the presence of a bromine atom. The molecular formula C_30_H_41_BrN_4_O_4_ of compound **4h** was confirmed by its molecular weight in combination with the data of elemental analysis. The IR spectrum of **4h** exhibited strong C–H vibration bands at 2926 and 2852 cm^−1^. A very strong absorption band at 1727 cm^−1^ was due to the C=O stretch vibrations of the methyl ester moiety. The ^1^H-NMR spectrum of **4h** showed three singlets at δ 1.30, 1.32 and 3.69 ppm, attributed to methyl protons at C-16, C-17 and 15-ester group, respectively. Two triplets, each containing four protons, at δ 2.55 and 2.60 ppm were due to eight protons at C-1′′ on two morpholine rings, while the multiplet at δ 3.68 ppm (partially overlapped with the singlet at δ 3.69 ppm) could be assigned to the eight protons at C-2′′. In addition, four doublets at δ 4.05, 4.10, 4.11 and 4.15 ppm could be attributed to the protons of the two methylenes at C-5′ and C-6′. The signals of the only aromatic proton at C-11 appeared as a singlet at δ 7.95 ppm. In the ^13^C-NMR spectrum of **4h**, there were 26 peaks appearing in the δ range from 16.4 to 178.8 ppm. Among them, two peaks at δ 55.1 and 55.7 ppm could be assigned to the four carbons at C-1′′ and two signals at δ 66.7 and 67.3 ppm were due to the four carbons at C-2′′, because the two morpholine rings presented different chemical shifts, but both of them were symmetrical structures. In addition, eight peaks at the δ 120.7, 128.8, 131.2, 138.3, 140.2, 150.6, 151.8 and 152.1 ppm were due to the aromatic carbons on the quinoxaline moiety. The peaks at δ 52.1 and 178.8 ppm could be attributed to the signals of methyl carbon and carbonyl carbon on the 15-ester group, respectively. The assignments of the signals in the ^1^H- and ^13^C-NMR spectra of **4h** were in good accordance with its structure.

### 2.2. Antiproliferative Effects of Dehydroabietic Acid (DAA) and Its Derivatives

All the compounds (**1**–**3**, **4a**–**o**) were evaluated for their in vitro cytotoxic activity against three cancer cell lines including human breast cancer cell line (MCF-7), human hepatocarcinoma cell line (SMMC-7721), human cervical carcinoma cell line (HeLa), and a noncancerous human hepatocyte cell line (LO2) using the MTT assay method [30]. An anticancer drug etoposide (VP-16) was co-assayed as the positive control. The results of the test compounds shown as IC_50_ values (concentration required to inhibit tumor cell proliferation by 50%), are listed in Table 1. As a result, the starting material, DAA (**1**), did not show cytotoxic activity against any tested cell line (IC_50_ > 50 μM), and intermediates **2** and **3** only showed mild cytotoxicity to HeLa and/or SMMC-7721 cell lines. On the other hand, most target compounds exhibited considerable anticancer activity against the three cancer cell lines. Among them, compounds **4a**–**c**, **4f**, **4i**, **4j** and **4m** showed strong cytotoxicity against at least one cancer cell line (IC_50_ < 10 μM). Compound **4b**, especially, exhibited the most potent activity against MCF-7, SMMC-7721 and HeLa cells, with IC_50_ values of 1.78 ± 0.36, 0.72 ± 0.09 and 1.08 ± 0.12 μM, respectively, equivalent to those of the positive control. Notably, the cytotoxicity of compound **4b** against normal human hepatocyte LO2 (IC_50_: 11.09 ± 0.57 μM) was substantially lower than that against the three cancer cells. On the other hand, compounds **4e**, **4k**, **4l**, **4n** and **4o** displayed moderate cytotoxic activity, while compounds **4d** and **4g** only showed weak activity against the three cancer cell lines.

As shown in Table 1, most target compounds (**4a**–**o**) exhibited stronger cytotoxic activity than compounds **1**–**3**, which indicated that the introduction of quinoxaline heterocycles and *N*-containing moieties on 2,3-side chains could considerably increase the cytotoxicity of DAA derivatives. On the other hand, the properties of the *N*-containing moiety, especially lipophilicity, played a crucial role for their activities. For example, compounds **4a** (CLogP = 4.69) and **4b** (CLogP = 6.81), with dimethylamino and diethylamino substituents exhibited stronger cytotoxic activity than **4c** (CLogP = 8.92) and **4d** (CLogP = 11.04) with more lipophilic dipropylamino and dibutylamino moieties, respectively. Concerning compounds **4e**–**k** with aliphatic *N*-containing rings, **4f** (CLogP = 7.08) with piperidine rings exhibited stronger cytotoxicity than **4g** (CLogP = 8.20) with azepane rings. In addition, the replacement of piperazine (**4i**, CLogP = 4.49) with *N*-methyl piperazine (**4j**, CLogP = 5.40) or *N*-ethyl piperazine (**4k**, CLogP = 6.58) led to a substantial decrease in cytotoxicity. These results indicated the introduction of aliphatic *N*-containing side chains with a larger size and lipophilicity would likely decrease the antitumor potencies. As for compounds **4l**–**o** with azole heterocycles, compound **4m** (CLogP = 3.21) containing 1,2,3-triazole rings obtained the strongest cytotoxicity. Additionally, compound **4n** (CLogP = 3.86) with 1*H*-tetrazol-1-yl moiety also possessed a superior cytotoxic activity, compared with its regioisomer **4o** (CLogP = 3.44). A possible explanation for these results was that the lipophilicity was not the only deciding factor for the anticancer activity of these derivatives. The number and position of nitrogen atoms on azole heterocycles might influence their physiochemical properties and the ability to interact with the potential targets in tumor cells, thereby affecting their anticancer activities.

### 2.3. Cell Cycle Analysis

**Figure 1:**
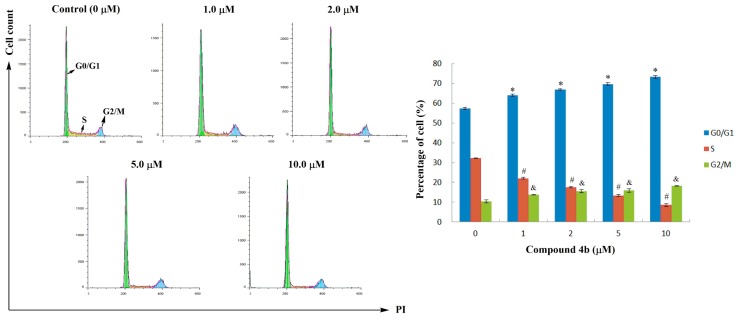
Effect of compound **4b** on the cell cycle distribution of human hepatocarcinoma SMMC- 7721 cells. * Percentage of G0/G1 phase cells in compound **4b**-treated groups, compared with the DMSO treated control group (*p*-value < 0.001). ^#^ Percentage of S phase cells in compound **4b**-treated groups, compared with the DMSO-treated control group (*p*-value < 0.001). ^&^ Percentage of G2/M phase cells in compound **4b**-treated groups, compared with the DMSO-treated control group (*p*-value < 0.001).

Subsequently, to determine the possible role of the cell cycle arrest in cell growth inhibition induced by target compounds, SMMC-7721 cells were treated with different concentrations of compound **4b**. The cell cycle distribution was investigated by flow cytometric analysis after staining of DNA with propidium iodide (PI). After treatment with compound **4b** at different concentrations (0, 1.0, 2.0, 5.0 and 10.0 μM) for 48 h, it was observed that S phase cells significantly decreased from 32.24 ± 0.21% to 8.54 ± 0.57%, while G0/G1 phase cells gradually increased from 57.34 ± 0.48% to 73.33 ± 0.69%. The ratio of G2/M phase cells also exhibited a small increase from 10.42 ± 0.68% to 18.13 ± 0.13% (Figure 1). These results suggested that target compound **4b** could arrest SMMC-7721 cells in the G0/G1 stage.

### 2.4. Hoechst 33258 Staining Assay

**Figure 2:**
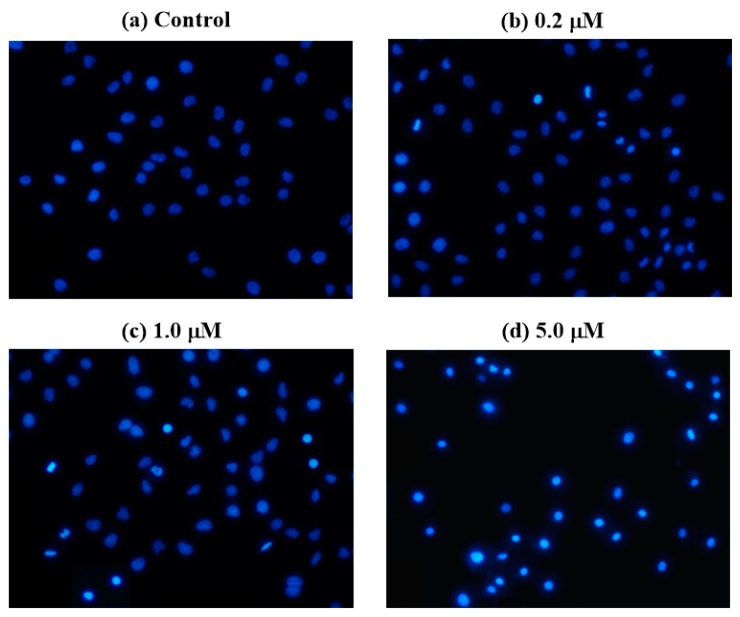
Effect of compound **4b** on nuclear morphological changes of SMMC-7721 cells.

To investigate the morphological changes induced by compound **4b** in SMMC-7721 cells, Hoechst staining was carried out. Hoechst 33258 is a cell membrane permeable dye, which stains the live cells’ nuclei uniformly as light-blue and apoptotic cells’ nuclei as round and bright-blue, on account of karyopyknosis and chromatin condensation [31,32]. SMMC-7721 cells were treated with various concentrations (0, 0.2, 1.0 and 5.0 μM) of compound **4b** for 24 h, and stained with Hoechst 33258. The results in Figure 2 showed that control cells had no obvious morphological changes; most cells’ nuclei appeared as uniformly ovoid light blue nuclei. In the 0.2 and 1.0 μM groups, part of the cells’ nuclei became irregularly shaped and some exhibited bright-blue fluorescence because of chromatin condensation, which is a typical characteristic of apoptosis. The number of apoptotic nuclei significantly increased along with the increase of the concentration of **4b** to 5.0 μM, which demonstrated that compound **4b** could induce apoptosis of SMMC-7721 cells in a dose-dependent manner.

### 2.5. Annexin V-FITC/PI Dual Staining Assay

**Figure 3:**
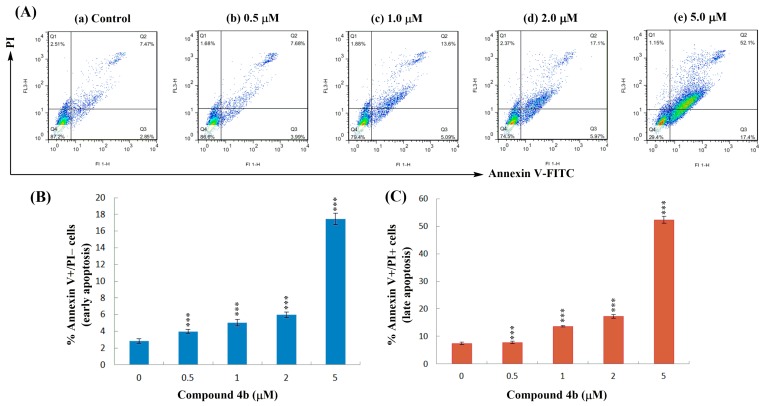
Annexin V-FITC/Propidium iodide (PI) dual staining assay of SMMC-7721 cells treated with compound **4b** using flow cytometry (**A**); Annexin V+/PI− indicated early phase of apoptosis (**B**); Annexin V+/PI+ indicated late apoptosis (**C**). *** *p* value < 0.001 for compound **4b** treatment, compared with the DMSO-treated control group.

The apoptotic effect of compound **4b** was further evaluated by a Annexin V-FITC/propidium iodide (AV/PI) dual staining assay to examine the occurrence of phosphatidylserine externalization, which facilitated the detection of live cells (lower left quadrant; AV−/PI−), early apoptotic cells (lower right quadrant; AV+/PI−), late apoptotic cells (upper right quadrant; AV+/PI+) and necrotic cells (upper left quadrant; AV−/PI+) [32]. As shown in Figure 3, the percentage of early apoptotic cells increased from 2.84 ± 0.26% (control) to 17.44 ± 0.68% (5.0 μM), and the percentage of late apoptotic cells also increased from 7.45 ± 0.42% (control) to 52.36 ± 1.23% (5.0 μM). The significant increase of apoptotic cells from 10.29% to 69.80% clearly indicated that compound **4b** could induce the apoptosis of SMMC-7721 cells in a dose-dependent manner.

## 3. Experimental Section

### 3.1. Materials and Methods

IR spectra were measured on a Nexus 870 FT-IR spectrometer (Thermo Nicolet Co. Ltd., Waltham, MA, USA), and the absorption bands were expressed in cm^−1^. The ESI-MS spectra were recorded on a Mariner System 5304 mass spectrometer (Thermo Fisher Scientific Inc., Waltham, MA, USA). ^1^H-NMR and ^13^C-NMR spectra were accomplished in CDCl_3_ on Bruker AV-300 and DRX-600 NMR spectrometers (Bruker Scientific Technology Co. Ltd., Karlsruhe, Germany), using TMS as the internal standard. Elemental analyses were carried out by the Elementar Vario El cube elemental analyzer. Reactions and the resulting products were monitored by TLC, which was carried out on silica gel IB-F flexible sheets from Mallinckrodt Baker Inc., Phillipsburg, NJ, USA and visualized in UV light (254 nm). Silica gel (300–400 mesh) for column chromatography was purchased from Qingdao Marine Chemical Factory, China. DAA (98%) was bought from Yijing Industrial Co., Ltd. (Shanghai, China). The reagents (chemicals), all being of AR grade, were purchased from the Shanghai Chemical Reagent Company (Shanghai, China).

### 3.2. Procedure for the Synthesis of Compound ***3***

The key intermediate (**2**) was synthesized from DAA (**1**) according to the procedure previously reported [29], which was further treated as follows to afford compounds **3** and **4** (Scheme 1). To a solution of compound **2** (0.130 g, 0.34 mmol) in 10 mL of acetic acid was added 0.3 mL (0.34 mmol) of 1,4-dibromobutane-2,3-dione. The mixture was refluxed at 120 °C for 2 h under a nitrogen atmosphere. After cooling, the mixture was poured into 100 mL of ice-cold water and extracted with EtOAc (3 × 60 mL). The organic layer was combined, washed with water, saturated NaHCO_3_ solution and brine, dried over anhydrous Na_2_SO_4_ and concentrated in vacuo to give a crude product, which was purified by silica gel column chromatography (petroleum ether-acetone; 20:1, *v*/*v*) to afford compound **3** (0.156 g, 78% yield). Yellow resin. IR (KBr, cm^−1^): 2926, 2853, 1726, 1588, 1540, 1457, 1248, 1186, 1028, 960. ^1^H-NMR (600 MHz, CDCl_3_): δ 1.30 (s, 3H), 1.32 (s, 3H), 1.50–1.90 (m, 7H), 2.30 (dd, *J* = 12.6, 1.8 Hz, 1H), 2.38 (d, *J* = 13.0 Hz, 1H), 3.18 (ddd, *J* = 19.1, 11.5, 7.7 Hz, 1H), 3.52 (dd, *J* = 18.8, 6.1 Hz, 1H), 3.70 (s, 3H, COOCH_3_), 4.94 (s, 2H), 4.97 (s, 2H), 8.02 (s, 1H). ^13^C-NMR (150 MHz, CDCl_3_): δ 16.4, 18.5, 21.1, 24.6, 25.6, 30.5, 30.7, 36.5, 37.9, 38.1, 44.9, 47.6, 52.2 (COO*C*H_3_), 120.9, 128.8, 130.9, 138.5, 140.2, 150.9, 151.5, 152.0, 178.8 (C=O). ESIMS: *m*/*z* 587.0 [M + H]^+^. Anal. Calcd. for C_22_H_25_Br_3_N_2_O_2_: C, 44.85; H, 4.28; N, 4.75. Found: C, 44.81; H, 4.32; N, 4.69.

### 3.3. General Procedures for the Synthesis of Compounds ***4a***–***o***

To a solution of compound **3** (0.295 g, 0.5 mmol) in 15 mL of acetonitrile was added K_2_CO_3_ (0.276 g, 2 mmol), KI (0.083 g, 0.5 mmol) and 10 mmol of different aliphatic amine or azole compounds. The mixture was refluxed for 8–12 h and the reaction was monitored by TLC. The mixture was then poured into cold water and extracted with CH_2_Cl_2_ (100 mL) three times. The organic phase was combined, washed with water and brine, dried over anhydrous Na_2_SO_4_ and concentrated in vacuo. The residue was subjected to silica gel chromatography (CH_2_Cl_2_–MeOH; 30:1 *v*/*v*) to afford compounds **4a**–**o**.

*Methyl 12-bromo-2′,3′-bis((dimethylamino)methyl)-13,14-pyrazinyldeisopropyl-dehydroabietate* (**4a**): yellow resin; 52% yield. IR (KBr, cm^−1^): 2924, 2854, 1726, 1616, 1464, 1383, 1248, 1124, 1034, 988. ^1^H-NMR (300 MHz, CDCl_3_): δ 1.31 (s, 3H), 1.33 (s, 3H), 1.50–1.90 (m, 7H), 2.10–2.40 (m, 2H), 2.40 (s, 6H), 2.47 (s, 6H), 3.20 (m, 1H), 3.56 (dd, *J* = 18.4, 6.0 Hz, 1H), 3.69 (s, 3H), 4.07 (brs, 2H), 4.14 (brs, 2H), 7.96 (s, 1H). ^13^C-NMR (150 MHz, CDCl_3_): δ 16.4, 18.5, 21.0, 24.6, 25.7, 36.4, 37.7, 38.2, 44.5, 45.6, 46.1, 47.6, 52.0 (COOCH_3_), 64.8, 65.1, 120.9, 128.8, 130.8, 138.6, 140.3, 150.9, 151.9, 152.2, 178.8 (C=O). ESIMS: *m*/*z* 517.2, 519.2 [M + H]^+^. Anal. Calcd. for C_26_H_37_BrN_4_O_2_: C, 60.34; H, 7.21; N, 10.83. Found: C, 60.38; H, 7.26; N, 10.78.

*Methyl 12-bromo-2′,3′-bis((diethylamino)methyl)-13,14-pyrazinyldeisopropyl-dehydroabietate* (**4b**): yellow resin; 62% yield. IR (KBr, cm^−1^): 2926, 2854, 1726, 1613, 1462, 1384, 1247, 1186, 1125, 1081, 966. ^1^H-NMR (300 MHz, CDCl_3_): δ 1.07 (t, *J* = 7.0 Hz, 6H), 1.11 (t, *J* = 7.1 Hz, 6H), 1.31 (s, 3H), 1.33 (s, 3H), 1.50–1.90 (m, 7H), 2.10–2.40 (m, 2H), 2.75 (q, *J* = 7.1 Hz, 4H), 2.87 (q, *J* = 6.8 Hz, 4H), 3.15 (m, 1H), 3.53 (dd, *J* = 19.0, 5.9 Hz, 1H), 3.70 (s, 3H), 4.22 (brs, 2H), 4.31 (brs, 2H), 7.95 (s, 1H). ^13^C-NMR (150 MHz, CDCl_3_): δ 10.9, 11.0, 16.4, 18.5, 21.1, 24.6, 25.5, 36.5, 37.9, 38.2, 44.9, 46.7, 47.1, 47.8, 52.1 (COO*C*H_3_), 59.5, 61.0, 120.9, 128.8, 130.9, 138.5, 140.3, 150.9, 151.7, 152.1, 178.8 (C=O). ESIMS: *m*/*z* 573.3, 575.3 [M + H]^+^. Anal. Calcd. for C_30_H_45_BrN_4_O_2_: C, 62.81; H, 7.91; N, 9.77. Found: C, 62.87; H, 7.88; N, 9.82.

*Methyl 12-bromo-2′,3′-bis((dipropylamino)methyl)-13,14-pyrazinyldeisopropyl-dehydroabietate* (**4c**): yellow resin; 48% yield. IR (KBr, cm^−1^): 2928, 2855, 1725, 1610, 1460, 1382, 1243, 1191, 1123, 1073, 987. ^1^H-NMR (300 MHz, CDCl_3_): δ 0.92 (t, *J* = 7.2 Hz, 6H), 0.96 (t, *J* = 7.1 Hz, 6H), 1.30 (s, 3H), 1.33 (s, 3H), 1.43–1.47 (m, 8H), 1.50–1.90 (m, 7H), 2.10–2.40 (m, 2H), 2.76 (q, *J* = 7.0 Hz, 4H), 2.89 (q, *J* = 6.9 Hz, 4H), 3.17 (m, 1H), 3.52 (dd, *J* = 18.7, 6.0 Hz, 1H), 3.68 (s, 3H), 4.12 (brs, 2H), 4.18 (brs, 2H), 7.93 (s, 1H). ^13^C-NMR (150 MHz, CDCl_3_): δ 11.2, 11.3, 16.4, 18.5, 21.0, 21.3, 21.5, 24.6, 25.7, 36.5, 37.8, 38.1, 45.0, 47.7, 52.0 (COOCH_3_), 58.6, 59.1, 61.3, 61.8, 120.9, 129.0, 130.8, 138.4, 140.2, 150.9, 151.7, 152.0, 178.9 (C=O). ESIMS: *m*/*z* 629.3, 631.3 [M + H]^+^. Anal. Calcd. for C_34_H_53_BrN_4_O_2_: C, 64.85; H, 8.48; N, 8.90. Found: C, 64.78; H, 8.52; N, 8.93.

*Methyl 12-bromo-2′,3′-bis((dibutylamino)methyl)-13,14-pyrazinyldeisopropyl-dehydroabietate* (**4d**): yellow resin; 52% yield. IR (KBr, cm^−1^): 2929, 2856, 1724, 1612, 1458, 1380, 1241, 1192, 1117, 1068, 977. ^1^H-NMR (300 MHz, CDCl_3_): δ 0.88 (t, *J* = 7.0 Hz, 6H), 0.93 (t, *J* = 7.1 Hz, 6H), 1.30 (s, 3H), 1.33 (s, 3H), 1.38–1.48 (m, 16H), 1.50–1.90 (m, 7H), 2.10–2.40 (m, 2H), 2.74 (q, *J* = 7.2 Hz, 4H), 2.86 (q, *J* = 6.8 Hz, 4H), 3.15 (m, 1H), 3.53 (dd, *J* = 18.7, 6.2 Hz, 1H), 3.71 (s, 3H), 4.09 (brs, 2H), 4.15 (brs, 2H), 7.95 (s, 1H). ^13^C-NMR (150 MHz, CDCl_3_): δ 13.2, 13.4, 16.5, 18.5, 20.4, 20.6, 21.1, 24.6, 25.5, 31.8, 32.1, 36.5, 37.7, 38.1, 44.9, 47.7, 52.0 (COOCH_3_), 54.6, 55.1, 60.2, 60.8, 120.7, 128.7, 130.8, 138.5, 140.2, 150.8, 151.6, 152.0, 179.0 (C=O). ESIMS: *m*/*z* 685.4, 687.4 [M + H]^+^. Anal. Calcd. for C_38_H_61_BrN_4_O_2_: C, 66.55; H, 8.97; N, 8.17. Found: C, 66.62; H, 9.01; N, 8.21.

*Methyl 12-bromo-2′,3′-bis(pyrrolidin-1-ylmethyl)-13,14-pyrazinyldeisopropyl-dehydroabietate* (**4e**): yellow resin; 57% yield. IR (KBr, cm^−1^): 2930, 2857, 1722, 1612, 1455, 1381, 1241, 1188, 1121, 1063, 985. ^1^H-NMR (300 MHz, CDCl_3_): δ 1.31 (s, 3H), 1.34 (s, 3H), 1.50–1.90 (m, 15H), 2.30 (d, *J* = 12.5 Hz, 1H), 2.37 (d, *J* = 12.7 Hz, 1H), 2.60 (brs, 4H), 2.64 (brs, 4H), 3.14 (m, 1H), 3.52 (dd, *J* = 18.8, 6.1 Hz, 1H), 3.69 (s, 3H), 4.03 (d, *J* = 13.0 Hz, 1H), 4.05 (d, *J* = 14.0 Hz, 1H), 4.09 (d, *J* = 10.4 Hz, 1H), 4.11 (d, *J* = 13.2 Hz, 1H), 7.93 (s, 1H). ^13^C-NMR (150 MHz, CDCl_3_): δ 16.4, 18.5, 21.1, 23.2, 23.6, 24.6, 25.6, 36.4, 37.8, 38.2, 44.9, 47.6, 51.9 (COOCH_3_), 56.7, 57.1, 59.2, 59.9, 120.8, 128.8, 130.9, 138.5, 140.2, 150.9, 151.6, 152.1, 178.9 (C=O). ESIMS: *m*/*z* 569.2, 571.2 [M + H]^+^. Anal. Calcd. for C_30_H_41_BrN_4_O_2_: C, 63.26; H, 7.26; N, 9.84. Found: C, 63.20; H, 7.31; N, 9.81.

*Methyl 12-bromo-2′,3′-bis(piperidin-1-ylmethyl)-13,14-pyrazinyldeisopropyl-dehydroabietate* (**4f**): yellow resin; 58% yield. IR (KBr, cm^−1^): 2931, 2853, 1728, 1591, 1463, 1383, 1247, 1125, 986. ^1^H-NMR (600 MHz, CDCl_3_): δ 1.31 (s, 3H), 1.34 (s, 3H), 1.40–1.47 (m, 4H), 1.50–1.56 (m, 9H), 1.61 (dd, *J* = 13.0, 7.9 Hz, 1H), 1.68 (dd, *J* = 9.5, 3.4 Hz, 1H), 1.75–1.90 (m, 4H), 2.30 (dd, *J* = 12.6, 1.9 Hz, 1H), 2.37 (d, *J* = 12.8 Hz, 1H), 2.47 (brs, 4H), 2.50 (brs, 4H), 3.16 (ddd, *J* = 19.0, 11.2, 7.8 Hz, 1H), 3.54 (dd, *J* = 18.9, 6.0 Hz, 1H), 3.69 (s, 3H, COOCH_3_), 4.03 (d, *J* = 13.0 Hz, 1H), 4.05 (d, *J* = 14.0 Hz, 1H), 4.07 (d, *J* = 11.4 Hz, 1H), 4.11 (d, *J* = 13.1 Hz, 1H), 7.92 (s, 1H). ^13^C-NMR (150 MHz, CDCl_3_): δ 16.4, 18.5, 21.1, 24.2, 24.5, 24.6, 25.6, 26.1, 26.5, 36.6, 37.9, 38.1, 45.1, 47.6, 51.9 (COOCH_3_), 55.2, 55.7, 59.8, 60.5, 120.8, 128.8, 130.9, 138.5, 140.2, 150.9, 151.8, 152.3, 178.9 (C=O). ESIMS: *m*/*z* 597.3, 599.3 [M + H]^+^. Anal. Calcd. for C_32_H_45_BrN_4_O_2_: C, 64.31; H, 7.59; N, 9.37. Found: C, 64.35; H, 7.62; N, 9.30.

*Methyl 2′,3′-bis(azepan-1-ylmethyl)-12-bromo-13,14-pyrazinyldeisopropyl-dehydroabietate* (**4g**): yellow resin; 57% yield. IR (KBr, cm^−1^): 2927, 2859, 1726, 1608, 1459, 1382, 1245, 1129, 1063, 971. ^1^H-NMR (300 MHz, CDCl_3_): δ 1.30 (s, 3H), 1.33 (s, 3H), 1.50–1.90 (m, 23H), 2.10–2.40 (m, 2H), 2.61 (brs, 4H), 2.72 (brs, 4H), 3.15 (m, 1H), 3.53 (dd, *J* = 18.8, 6.3 Hz, 1H), 3.70 (s, 3H), 4.06 (brs, 2H), 4.12 (brs, 2H), 7.92 (s, 1H). ^13^C-NMR (150 MHz, CDCl_3_): δ 16.4, 18.5, 21.0, 24.6, 25.5, 25.9, 26.4, 27.5, 28.1, 36.5, 37.9, 38.2, 44.9, 47.6, 52.1 (COOCH_3_), 60.2, 61.0, 120.8, 128.9, 130.9, 138.6, 140.5, 150.8, 151.7, 152.3, 178.8 (C=O). ESIMS: *m*/*z* 625.3, 627.3 [M + H]^+^. Anal. Calcd. for C_34_H_49_BrN_4_O_2_: C, 65.27; H, 7.89; N, 8.95. Found: C, 65.33; H, 7.93; N, 8.89.

*Methyl 12-bromo-2′,3′-bis(morpholinomethyl)-13,14-pyrazinyldeisopropyl-dehydroabietate* (**4h**): yellow resin; 63% yield. IR (KBr, cm^−1^): 2926, 2852, 1727, 1615, 1453, 1382, 1324, 1247, 1119, 1007, 865. ^1^H-NMR (600 MHz, CDCl_3_): δ 1.30 (s, 3H), 1.32 (s, 3H), 1.54 (dt, *J* = 12.6, 3.1 Hz, 1H), 1.62 (dd, *J* = 12.8, 8.0 Hz, 1H), 1.70 (dd, *J* = 12.7, 3.7 Hz, 1H), 1.75–1.90 (m, 4H), 2.30 (dd, *J* = 12.5, 1.8 Hz, 1H), 2.38 (d, *J* = 12.9 Hz, 1H), 2.55 (t, *J* = 4.1 Hz, 4H), 2.60 (t, *J* = 4.2 Hz, 4H), 3.16 (ddd, *J* = 18.9, 11.2, 7.4 Hz, 1H), 3.53 (dd, *J* = 18.7, 6.1 Hz, 1H), 3.68 (m, 8H), 3.69 (s, 3H, COOCH_3_), 4.05 (d, *J* = 13.0 Hz, 1H), 4.10 (d, *J* = 13.0 Hz, 1H), 4.11 (d, *J* = 13.3 Hz, 1H), 4.15 (d, *J* = 13.1 Hz, 1H), 7.95 (s, 1H). ^13^C-NMR (150 MHz, CDCl_3_): δ 16.4, 18.5, 20.9, 24.6, 25.5, 36.6, 37.8, 38.2, 45.0, 47.7, 52.0 (COOCH_3_), 55.1 (2 × CH_2_), 55.7 (2 × CH_2_), 60.2, 60.9, 66.7 (2 × CH_2_), 67.3 (2 × CH_2_), 120.7, 128.8, 131.2, 138.3, 140.2, 150.6, 151.8, 152.1, 178.8 (C=O). ESIMS: *m*/*z* 601.2, 603.2 [M + H]^+^. Anal. Calcd. for C_30_H_41_BrN_4_O_4_: C, 59.90; H, 6.87; N, 9.31. Found: C, 59.82; H, 6.92; N, 9.38.

*Methyl 12-bromo-2′,3′-bis(piperazin-1-ylmethyl)-13,14-pyrazinyldeisopropyl-dehydroabietate* (**4i**): yellow resin; 53% yield. IR (KBr, cm^−1^): 2928, 2847, 2797, 1725, 1590, 1453, 1379, 1242, 1152, 1018, 857. ^1^H-NMR (300 MHz, CDCl_3_): δ 1.30 (s, 3H), 1.34 (s, 3H), 1.50–1.90 (m, 7H), 2.10–2.40 (m, 2H), 2.40 (brs, 2H, NH), 2.46 (brs, 8H), 2.84 (brs, 4H), 2.89 (brs, 4H), 3.16 (ddd, *J* = 19.0, 11.2, 7.3 Hz, 1H), 3.53 (dd, *J* = 18.8, 6.3 Hz, 1H), 3.68 (s, 3H), 4.09 (brs, 2H), 4.15 (brs, 2H), 7.97 (s, 1H). ^13^C-NMR (150 MHz, CDCl_3_): δ 16.4, 18.5, 21.1, 24.6, 25.5, 36.6, 37.8, 38.2, 45.0, 46.2 (2 × CH_2_), 46.6 (2 × CH_2_), 47.8, 52.0 (COOCH_3_), 54.5 (2 × CH_2_), 55.1 (2 × CH_2_), 59.6, 60.1, 120.8, 128.7, 130.9, 138.5, 140.2, 150.9, 151.9, 152.2, 178.8 (C=O). ESIMS: *m*/*z* 599.3, 601.3 [M + H]^+^. Anal. Calcd. for C_30_H_43_BrN_6_O_2_: C, 60.09; H, 7.23; N, 14.02. Found: C, 60.15; H, 7.27; N, 13.96.

*Methyl 12-bromo-2′,3′-bis((4-methylpiperazin-1-yl)methyl)-13,14-pyrazinyl-deisopropyl-dehydroabietate* (**4j**): yellow resin; 49% yield. IR (KBr, cm^−1^): 2930, 2849, 2796, 1727, 1592, 1456, 1372, 1247, 1162, 1010, 814. ^1^H-NMR (300 MHz, CDCl_3_): δ 1.30 (s, 3H), 1.33 (s, 3H), 1.50–1.90 (m, 7H), 2.10–2.40 (m, 2H), 2.31 (s, 3H), 2.32 (s, 3H), 2.48 (brs, 8H), 2.58 (brs, 4H), 2.65 (brs, 4H), 3.17 (ddd, *J* = 18.8, 11.0, 7.5 Hz, 1H), 3.55 (dd, *J* = 18.7, 6.2 Hz, 1H), 3.70 (s, 3H), 4.08 (brs, 2H), 4.13 (brs, 2H), 7.95 (s, 1H). ^13^C-NMR (150 MHz, CDCl_3_): δ 16.4, 18.5, 21.1, 24.6, 25.6, 36.6, 37.9, 38.4, 45.0, 46.5 (N-CH_3_), 46.8 (N-CH_3_), 47.7, 52.2 (COOCH_3_), 53.8 (2 × CH_2_), 54.2 (2 × CH_2_), 56.7 (2 × CH_2_), 57.2 (2 × CH_2_), 59.8, 60.6, 120.8, 128.9, 130.9, 138.6, 140.2, 150.8, 151.9, 152.3, 178.9 (C=O). ESIMS: *m*/*z* 627.3, 629.3 [M + H]^+^. Anal. Calcd. for C_32_H_47_BrN_6_O_2_: C, 61.23; H, 7.55; N, 13.39. Found: C, 61.17; H, 7.58; N, 13.32.

*Methyl 12-bromo-2′,3′-bis((4-ethylpiperazin-1-yl)methyl)-13,14-pyrazinyl-deisopropyl- dehydroabietate* (**4k**): yellow resin; 40% yield. IR (KBr, cm^−1^): 2926, 2852, 2812, 1728, 1667, 1594, 1462, 1380, 1246, 1125, 966. ^1^H-NMR (300 MHz, CDCl_3_): δ 1.11 (t, *J* = 7.0 Hz, 3H), 1.13 (t, *J* = 7.1 Hz, 3H), 1.29 (s, 3H), 1.32 (s, 3H), 1.50–1.90 (m, 7H), 2.10–2.40 (m, 2H), 2.48 (m, 8H), 2.51 (m, 4H), 2.63 (brs, 4H), 2.71 (brs, 4H), 3.15 (ddd, *J* = 18.8, 11.2, 8.0 Hz, 1H), 3.53 (dd, *J* = 18.8, 6.0 Hz, 1H), 3.69 (s, 3H), 4.06 (brs, 2H), 4.13 (brs, 2H), 7.94 (s,1H ). ^13^C-NMR (150 MHz, CDCl_3_): δ 12.8 (NCH_2_*C*H_3_), 13.1 (NCH_2_***C***H_3_), 16.4, 18.5, 21.1, 24.6, 25.5, 36.5, 37.9, 38.3, 45.0, 47.6, 50.2 (NCH_2_), 50.6 (NCH_2_), 52.1 (COOCH_3_), 54.8 (2 × CH_2_), 55.2 (2 × CH_2_), 57.2 (2 × CH_2_), 57.9 (2 × CH_2_), 60.7, 61.5, 120.9, 129.0, 131.0, 138.5, 140.2, 150.9, 151.9, 152.3, 179.0 (C=O). ESIMS: *m*/*z* 655.3, 657.3 [M + H]^+^. Anal. Calcd. for C_34_H_51_BrN_6_O_2_: C, 62.28; H, 7.84; N, 12.82. Found: C, 62.36; H, 7.88; N, 12.76.

*Methyl 12-bromo-2′,3′-bis((1H-imidazol-1-yl)methyl)-13,14-pyrazinyl-deisopropyl-dehydroabietate* (**4l**): yellow resin; 46% yield. IR (KBr, cm^−1^): 2924, 2854, 1720, 1594, 1462, 1363, 1249, 1188, 1080, 969. ^1^H-NMR (300 MHz, CDCl_3_): δ 1.28 (s, 3H), 1.31 (s, 3H), 1.50–1.90 (m, 7H), 2.24 (d, *J* = 11.2 Hz, 1H), 2.34 (d, *J* = 12.7 Hz, 1H), 2.97 (m, 1H), 3.23 (dd, *J* = 18.8, 6.0 Hz, 1H), 3.70 (s, 3H), 5.65 (s, 2H), 5.71 (s, 2H), 6.96 (m, 2H), 7.16 (m, 2H), 7.73 (s, 1H), 7.78 (s, 1H), 7.95 (s, 1H). ^13^C-NMR (150 MHz, CDCl_3_): δ 16.5 (4-CH_3_), 18.5, 21.2, 24.6 (10-CH_3_), 25.7, 36.5, 37.9, 38.1, 45.1, 47.6, 52.2 (COOCH_3_), 50.5, 51.1, 118.5, 118.8, 120.8, 128.7, 129.8, 130.1, 130.9, 138.4, 139.3, 139.6, 140.2, 150.8, 151.7, 152.0, 178.9 (C=O). ESIMS: *m*/*z* 563.2, 565.2 [M + H]^+^. Anal. Calcd. for C_28_H_31_BrN_6_O_2_: C, 59.68; H, 5.55; N, 14.91. Found: C, 59.73; H, 5.52; N, 14.95.

*Methyl 12-bromo-2′,3′-bis((1H-1,2,3-triazol-1-yl)methyl)-13,14-pyrazinyl-deisopropyl-dehydroabietate* (**4m**): yellow resin; 41% yield. IR (KBr, cm^−1^): 2926, 2852, 1725, 1598, 1455, 1376, 1242, 1136, 1028, 985. ^1^H-NMR (300 MHz, CDCl_3_): δ 1.28 (s, 3H), 1.32 (s, 3H), 1.50–1.90 (m, 7H), 2.22 (d, *J* = 11.5 Hz, 1H), 2.34 (d, *J* = 12.6 Hz, 1H), 2.97 (m, 1H), 3.23 (dd, *J* = 18.9, 6.3 Hz, 1H), 3.72 (s, 3H), 5.69 (s, 2H), 5.73 (s, 2H), 7.76 (brs, 2H), 7.92 (brs, 2H), 8.04 (s, 1H). ^13^C-NMR (150 MHz, CDCl_3_): δ 16.4, 18.5, 21.2, 24.6, 25.7, 36.6, 37.9, 38.2, 45.1, 47.6, 51.9 (COOCH_3_), 52.2, 52.6, 120.8, 123.2, 123.6, 128.9, 130.9, 133.5, 133.9, 138.7, 140.2, 150.8, 151.9, 152.5, 178.9 (C=O). ESIMS: *m*/*z* 565.2, 567.2 [M + H]^+^. Anal. Calcd. for C_26_H_29_BrN_8_O_2_: C, 55.23; H, 5.17; N, 19.82. Found: C, 55.28; H, 5.11; N, 19.89.

*Methyl 12-bromo-2′,3′-bis((1H-tetrazol-1-yl)methyl)-13,14-pyrazinyl-deisopropyl-dehydroabietate* (**4n**): yellow resin; 35% yield; IR (KBr, cm^−1^): 2924, 2872, 1720, 1663, 1593, 1470, 1386, 1248, 1124, 1023, 958. ^1^H-NMR (300 MHz, CDCl_3_): δ 1.26 (s, 3H), 1.30 (s, 3H), 1.50–1.90 (m, 7H), 2.23 (d, *J* = 11.1 Hz, 1H), 2.34 (d, *J* = 12.6 Hz, 1H), 2.98 (ddd, *J* = 18.6, 11.0, 7.8 Hz, 1H), 3.23 (dd, *J* = 19.2, 6.3 Hz, 1H), 3.70 (s, 3H), 6.20 (s, 2H), 6.22 (s, 2H), 8.07 (s, 1H), 8.94 (s, 1H), 9.13 (s, 1H). ^13^C-NMR (150 MHz, CDCl_3_): δ 16.4, 18.5, 21.1, 24.6, 25.7, 36.6, 37.9, 38.2, 45.1, 47.7, 50.1, 50.5, 51.9 (COOCH_3_), 120.9, 128.7, 130.9, 138.3, 140.1, 145.2, 145.9, 150.9, 151.5, 151.8, 178.9 (C=O). ESIMS: *m*/*z* 567.2, 569.2 [M + H]^+^. Anal. Calcd. for C_24_H_27_BrN_10_O_2_: C, 50.80; H, 4.80; N, 24.68. Found: C, 50.72; H, 4.83; N, 24.75.

*Methyl 12-bromo-2′, 3′-bis((2H-tetrazol-2-yl)methyl)-13,14-pyrazinyl-deisopropyl-dehydroabietate* (**4o**): yellow resin; 32% yield. IR (KBr, cm^−1^): 2925, 2849, 1717, 1669, 1466, 1433, 1385, 1253, 1169, 1099, 964. ^1^H-NMR (300 MHz, CDCl_3_): δ 1.26 (s, 3H), 1.30 (s, 3H), 1.50–1.90 (m, 7H), 2.24 (dd, *J* = 12.3, 5.1 Hz, 1H), 2.35 (d, *J* = 12.3 Hz, 1H), 2.98 (m, 1H), 3.34 (dd, *J* = 19.2, 6.6 Hz, 1H), 3.71 (s, 3H), 6.41 (s, 2H), 6.42 (s, 2H), 8.06 (s, 1H), 8.60 (brs, 2H). ^13^C-NMR (150 MHz, CDCl_3_): δ 16.4, 18.5, 21.1, 24.6, 25.7, 36.6, 37.9, 38.3, 45.0, 47.7, 52.0 (COOCH_3_), 64.5, 65.1, 120.9, 128.8, 130.9, 138.6, 140.3, 150.9, 151.8, 152.1, 152.3, 152.6, 178.9 (C=O). ESIMS: *m*/*z* 567.2, 569.2 [M + H]^+^. Anal. Calcd. for C_24_H_27_BrN_10_O_2_: C, 50.80; H, 4.80; N, 24.68. Found: C, 50.86; H, 4.77; N, 24.62.

### 3.4. Cytotoxic Assay

The in vitro cytotoxic activities of the quinoxaline derivatives of DAA were evaluated against a human breast cancer cell line (MCF-7), human hepatocarcinoma cell line (SMMC-7721), human cervical carcinoma cell line (HeLa) and noncancerous human hepatocyte cells (LO2) via the MTT colorimetric method. Briefly, different tumor cells were grown in DMEM supplemented with 10% fetal bovine serum, penicillin (100 U/mL), and streptomycin (50 μg/mL). Cells were harvested at the log phase of growth and seeded in 96-well plates (100 μL/well at a density of 2 × 10^5^ cells/mL). After 24 h of incubation at 37 °C and 5% CO_2_ to allow for cell attachment, cultures were exposed to various concentrations of the isolated compounds for 48 h. Finally, the MTT solution (2.5 mg/mL in PBS) was added (40 μL/well). Plates were further incubated for 4 h at 37 °C, and the formazan crystals formed were dissolved by adding 150 μL/well of DMSO. Absorption at 570 nm was measured with an ELISA plate reader. The results were expressed as IC_50_ values with standard deviations, which were defined as the concentrations at which a 50% survival of the cells was discerned. Etoposide (VP-16) was co-assayed as the positive control.

### 3.5. Cell Cycle Analysis

SMMC-7721 cells were treated with indicated concentrations of compound **4b**. After incubation for 48 h, the cells were washed twice with ice-cold PBS, fixed and permeabilized with ice-cold 70% ethanol at −20 °C overnight. The cells were treated with 100 μg/mL RNase A at 37 °C for 30 min after being washed with ice-cold PBS, and finally stained with 400 μL of 1 mg/mL PI in the dark at 4 °C for 30 min. The samples were then analyzed for their DNA content by flow cytometry (Becton-Dickinson FACSCalibur, New York, NY, USA).

### 3.6. Hoechst 33258 Staining Assay

Cells grown on a sterile cover slip in six-well plates were treated with different concentrations of the test compound for 24 h. The culture medium containing compounds was removed, and the cells were fixed in 4% paraformaldehyde for 10 min. After being washed twice with PBS, the cells were stained with 0.5 mL of Hoechst 33258 (Beyotime, Haimen, China) for 5 min, and then again washed twice with PBS. The stained nuclei were observed under a Nikon ECLIPSETE2000-S fluorescence microscope using 350 nm for excitation and 460 nm for emission.

### 3.7. Annexin V-FITC/PI Dual Staining Assay

Apoptosis was discriminated with the Annexin V-FITC/PI dual staining assay. SMMC-7721 cells were seeded at 1 × 10^5^ cells per well in 10% fetal calf serum (FBS)-DMEM into six-well plates, and treated with compound **4b** for 24 h. The cells were washed twice with cold PBS and then resuspended in 1 × binding buffer (0.1 M Hepes/NaOH (pH 7.4), 1.4 M NaCl, 25 mM CaCl_2_) at a concentration of 1 × 10^6^ cells/mL. The solution (100 μL) was transferred to 5 mL culture tubes, and 5 μL of Annexin V-FITC (BD, Pharmingen) and 5 μL of PI were added to each tube. The cells were gently vortexed, and incubated for 30 min at 25 °C in the dark. PBS (200 μL) was added to each tube. The analysis was performed on a flow cytometry (Becton-Dickinson FACSCalibur, New York, NY, USA).

### 3.8. Statistical Analysis

All data from the three independent experiments were used for measuring the means ± standard error (mean ± S.E.) that were compared using the Student’s *t*-test. A value of *p* < 0.05 was considered statistically significant.

## 4. Conclusions

In summary, a series of new quinoxaline derivatives of DAA (**4a**–**o**) were designed, synthesized and evaluated for their in vitro cytotoxic activities against the three human cancer cell lines. As a result, a number of compounds exhibited pronounced antitumor activities. Among them, compound **4b** showed the most potent activity against the three cancer cell lines (MCF-7, SMMC-7721 and HeLa), comparable to the positive control etoposide and with considerably lower cytotoxicity to noncancerous human hepatocytes (LO2). In addition, compound **4b** caused cell cycle arrest at the G0/G1 phase and induced the apoptosis of SMMC-7721 cells in a dose-dependent manner. Therefore, this class of compounds can be considered as promising lead molecules for the development of more potent anticancer agents. Further research will also be carried out to investigate the in-depth structure–activity relationships and the anticancer mechanisms of these derivatives.

## Figures and Tables

**Table 1:** IC_50_ values of the synthesized compounds against four tested cell lines.

Compound	CLogP	IC_50_ (μM)			
		MCF-7	SMMC-7721	HeLa	LO2
**1**	−	>50	>50	>50	>50
**2**	−	>50	>50	43.32 ± 3.26	>50
**3**	−	>50	45.83 ± 5.28	37.72 ± 3.75	>50
**4a**	4.69	2.36 ± 0.29	1.65 ± 0.22	2.08 ± 0.43	15.72 ± 0.65
**4b**	6.81	1.78 ± 0.36	0.72 ± 0.09	1.08 ± 0.12	11.09 ± 0.57
**4c**	8.92	21.02 ± 1.27	10.96 ± 0.58	8.67 ± 0.65	>50
**4d**	11.04	28.13 ± 0.92	32.70 ± 2.69	25.32 ± 3.01	>50
**4e**	5.96	20.45 ± 1.51	18.64 ± 2.37	12.79 ± 1.26	>50
**4f**	7.08	8.95 ± 0.63	7.71 ± 0.82	5.98 ± 0.37	42.23 ± 2.78
**4g**	8.20	>50	27.89 ± 2.91	32.01 ± 4.21	>50
**4h**	4.52	12.32 ± 1.03	16.28 ± 1.85	9.67 ± 1.13	36.64 ± 3.12
**4i**	4.49	5.89 ± 0.56	4.32 ± 0.64	5.05 ± 0.72	21.95 ± 2.70
**4j**	5.40	12.21 ± 0.75	8.03 ± 0.70	6.62 ± 0.59	29.52 ± 3.61
**4k**	6.58	38.66 ± 2.87	26.45 ± 3.73	13.51 ± 2.21	>50
**4l**	3.75	22.84 ± 3.21	15.30 ± 2.06	12.73 ± 1.35	>50
**4m**	3.21	3.73 ± 0.62	4.66 ± 0.42	2.18 ± 0.37	18.36 ± 1.73
**4n**	3.86	15.76 ± 0.81	10.88 ± 1.68	13.26 ± 2.71	>50
**4o**	3.44	24.38 ± 1.69	16.92 ± 1.95	20.46 ± 3.12	>50
Etoposide	−	0.73 ± 0.07	0.69 ± 0.18	0.87 ± 0.16	8.89 ± 0.38

## Supplementary Materials

Supplementary File 1

## References

[1] Siegel R.L., Miller K.D., Jemal A. (2016). Cancer statistics, 2016. CA Cancer J. Clin..

[2] Zhang Q., Zhai S., Li L., Li X., Zhou H., Liu A., Su G., Mu Q., Du Y., Yan B. (2013). Anti-tumor selectivity of a novel tubulin and HSP90 dual-targeting inhibitor in non-small cell lung cancer models. Biochem. Pharmacol..

[3] Son K.H., Oh H.M., Choi S.K., Han D.C., Kwon B.M. (2005). Anti-tumor abietane diterpenes from the cones of *Sequoia sempervirens*. Bioorg. Med. Chem. Lett..

[4] Huang X.C., Huang R.Z., Liao Z.X., Pan Y.M., Gou S.H., Wang H.S. (2016). Synthesis and pharmacological evaluation of dehydroabietic acid thiourea derivatives containing bisphosphonate moiety as an inducer of apoptosis. Eur. J. Med. Chem..

[5] Newman D.J., Cragg G.M. (2012). Natural products as sources of new drugs over the 30 years from 1981 to 2010. J. Nat. Prod..

[6] Leandro L.F., Cardoso M.J., Silva S.D., Souza M.G., Veneziani R.C., Ambrosio S.R., Martins C.H. (2014). Antibacterial activity of *Pinus elliottii* and its major compound, dehydroabietic acid, against multidrug-resistant strains. J. Med. Microbiol..

[7] Chen N.Y., Duan W.G., Liu L.Z., Li F.Y., Lu M.P., Liu B.M. (2015). Synthesis and antifungal activity of dehydroabietic acid-based thiadiazole-phosphonates. Holzforschung.

[8] Li W.S., McChesney J.D. (1992). Preparation of potential anti-inflammatory agents from dehydroabietic dehydroabietic acid. J. Pharm. Sci..

[9] Sepulveda B., Astudillo L., Rodriguez J.A., Yanez T., Theoduloz C., Schmeda-Hirschmann G. (2005). Gastroprotective and cytotoxic effect of dehydroabietic acid derivatives. Pharmacol. Res..

[10] Zapata B., Rojas A., Betancur-Galvis L., Mesa-Arango A.C., Pérez-Guaitac D., González M.A. (2013). Cytotoxic, immunomodulatory, antimycotic, and antiviral activities of semisynthetic 14-hydroxyabietane derivatives and triptoquinone C-4 epimers. MedChemComm.

[11] Roa-Linares V.C., Brand Y.M., Agudelo-Gomez L.S., Tangarife-Castaño V., Betancur-Galvis L.A., Gallego-Gomez J.C., González M.A. (2016). Anti-herpetic and anti-dengue activity of abietane ferruginol analogues synthesized from (+)-dehydroabietylamine. Eur. J. Med. Chem..

[12] Kim J., Kang Y.G., Lee J.Y., Choi D.H., Cho Y.U., Shin J.M., Park J.S., Lee J.H., Kim W.G., Seo D.B. (2015). The natural phytochemical dehydroabietic acid is an anti-aging reagent that mediates the direct activation of SIRT1. Mol. Cell. Endocrinol..

[13] Fei B.L., Xu W.S., Gao W.L., Zhang J., Zhao Y., Long J.Y., Anson C.E., Powell A.K. (2015). DNA binding and cytotoxicity activity of a chiral iron(III) triangle complex based on a natural rosin product. J. Photochem. Photobiol. B.

[14] Jin L., Qu H.E., Huang X.C., Pan Y.M., Liang D., Chen Z.F., Wang H.S., Zhang Y. (2015). Synthesis and biological evaluation of novel dehydroabietic acid derivatives conjugated with acyl-thiourea peptide moiety as antitumor agents. Int. J. Mol. Sci..

[15] Luo D.J., Ni Q., Ji A.L., Gu W., Wu J.H., Jiang C.P. (2016). Dehydroabietic acid derivative QC4 induces gastric cancer cell death via oncosis and apoptosis. BioMed Res. Int..

[16] Wagner B., Schumann D., Linne U., Koert U., Marahiel M.A. (2006). Rational design of bacitracin A derivatives by incorporating natural product derived heterocycles. J. Am. Chem. Soc..

[17] Islam M.A., Zhang Y.Q., Wang Y., McAlpine S.R. (2015). Design, synthesis and anticancer mechanistic studies of linked azoles. MedChemComm.

[18] Gomtsyan A. (2012). Heterocycles in drugs and drug discovery. Chem. Heterocycl. Compd..

[19] Xu F.F., Cheng G.Y., Hao H.H., Wang Y.L., Wang X., Chen D.M., Peng D.P., Liu Z.L., Yuan Z.H., Dai M.H. (2016). Mechanisms of antibacterial action of quinoxaline 1,4-di-*N*-oxides against *Clostridium perfringens* and *Brachyspira hyodysenteriae*. Front. Microbiol..

[20] Loughran H.M., Han Z.Y., Wrobel J.E., Decker S.E., Ruthel G., Freedman B.D., Harty R.N., Reitz A.B. (2016). Quinoxaline-based inhibitors of Ebola and Marburg VP40 egress. Bioorg. Med. Chem. Lett..

[21] Cerecetto H., Dias E., Di Maio R., González M., Pacce S., Saenz P., Seoane G., Suescun L., Mombrú A., Fernández G. (2000). Synthesis and herbicidal activity of *N*-oxide derivatives. J. Agric. Food Chem..

[22] Guirado A., Sánchez J.I.L., Ruiz-Alcaraz A.J., Bautista D., Galvez J. (2012). Synthesis and biological evaluation of 4-alkoxy-6,9-dichloro[1,2,4]triazolo[4,3-a]quinoxalines as inhibitors of TNF-α and IL-6. Eur. J. Med. Chem..

[23] Zghaib Z., Guichou J.F., Vappiani J., Bec N., Hadj-Kaddour K., Vincent L.A., Paniagua-Gayraud S., Larroque C., Moarbess G., Cuq P. (2016). New imidazoquinoxaline derivatives: Synthesis, biological evaluation on melanoma, effect on tubulin polymerization and structure–activity relationships. Bioorg. Med. Chem..

[24] Corona P., Carta A., Loriga M., Vitale G., Paglietti G. (2009). Synthesis and in vitro antitumor activity of new quinoxaline derivatives. Eur. J. Med. Chem..

[25] Ghattass K., El-Sitt S., Zibara K., Rayes S., Haddadin M.J., El-Sabban M., Gali-Muhtasib H. (2014). The quinoxaline di-*N*-oxide DCQ blocks breast cancer metastasis in vitro and in vivo by targeting the hypoxia inducible factor-1 pathway. Mol. Cancer.

[26] Nagender P., Kumar R.N., Reddy G.M., Swaroop D.K., Poornachandra Y., Kumar C.G., Narsaiah B. (2016). Synthesis of novel hydrazone and azole functionalized pyrazolo[3,4-b]pyridine derivatives as promising anticancer agents. Bioorg. Med. Chem. Lett..

[27] Mareddy J., Suresh N., Kumar C.G., Kapavarapu R., Jayasree A., Pal S. (2017). 1,2,3-Triazole-nimesulide hybrid: Their design, synthesis and evaluation as potential anticancer agents. Bioorg. Med. Chem. Lett..

[28] Ma C.M., Cao R.H., Shi B.X., Zhou X.T., Ma Q., Sun J., Guo L., Yi W., Chen Z.Y., Song H.C. (2010). Synthesis and cytotoxic evaluation of 1-carboxamide and 1-amino side chain substituted β-carbolines. Eur. J. Med. Chem..

[29] Fonseca T., Gigante B., Marques M.M., Gilchrist T.L., de Clercq E. (2004). Synthesis and antiviral evaluation of benzimidazoles, quinoxalines and indoles from dehydroabietic acid. Bioorg. Med. Chem..

[30] Wang C.J., Delcros J.G., Biggerstaff J., Phanstiel O. (2003). Synthesis and biological evaluation of *N*-(anthracen-9-ylmethyl)triamines as molecular recognition elements for the polyamine transporter. J. Med. Chem..

[31] Wyllie A.H. (1980). Glucocorticoid-induced thymocyte apoptosis is associated with endogenous endonuclease activation. Nature.

[32] Reddy V.G., Reddy T.S., Nayak V.L., Prasad B., Reddy A.P., Ravikumar A., Taj S., Kamal A. (2016). Design, synthesis and biological evaluation of *N*-((1-benzyl-1*H*-1,2,3-triazol-4-yl)methyl)-1,3-diphenyl-1*H*-pyrazole-4-carboxamides as CDK1/Cdc2 inhibitors. Eur. J. Med. Chem..

