# Supplementary material for: Synthesis and Evaluation of New Quinoxaline Derivatives of Dehydroabietic Acid as Potential Antitumor Agents
Source: Molecules. 2017 Jul 11;22(7):1154. doi: 10.3390/molecules22071154 (PMC6152277; doi:10.3390/molecules22071154)
Supplement: Supplementary File 1 [file molecules-22-01154-s001.pdf]

Suplementary Materials

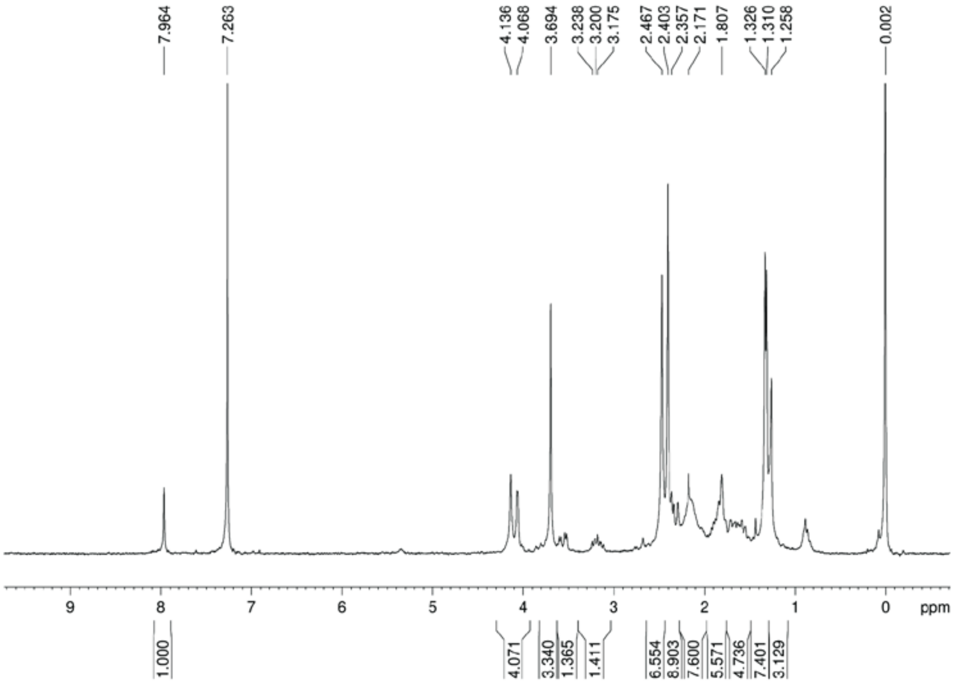

Figure S1. ^{1}H-NMR spectrum of compound **4a** (300 MHz, $CDCl_3$)

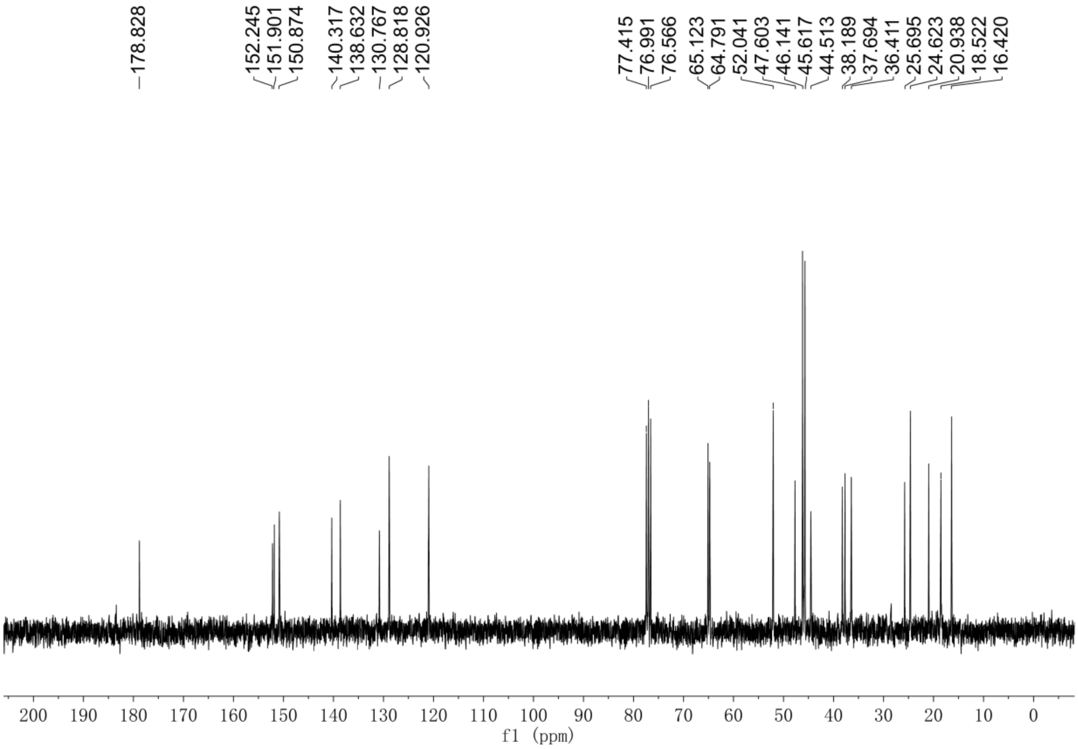

Figure S1. ^{13}C-NMR spectrum of compound **4a** (75 MHz, $CDCl_3$)

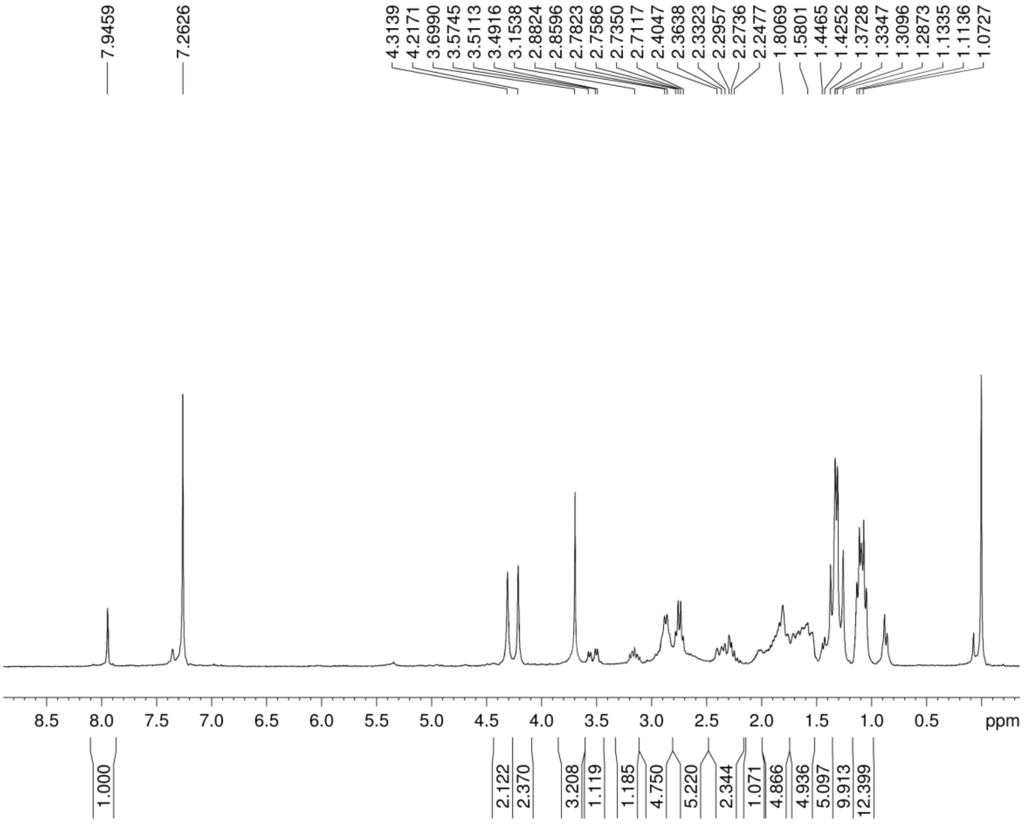

Figure S3. ^{1}H-NMR spectrum of compound **4b** (300 MHz, $CDCl_3$)

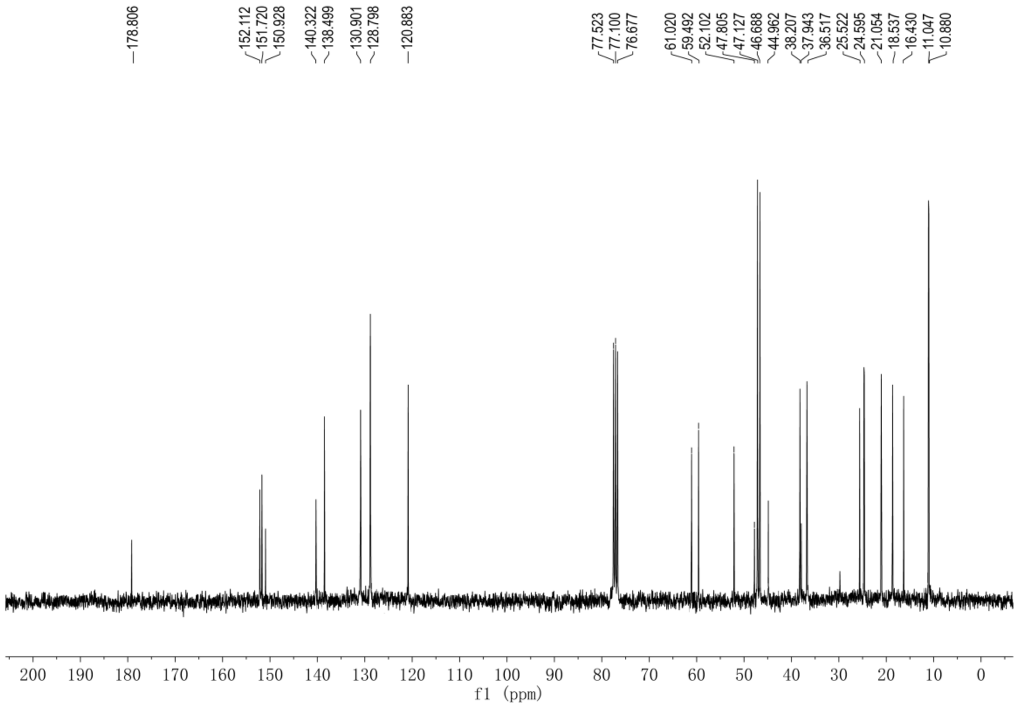

Figure S4. ^{13}C-NMR spectrum of compound **4b** (75 MHz, $CDCl_3$)

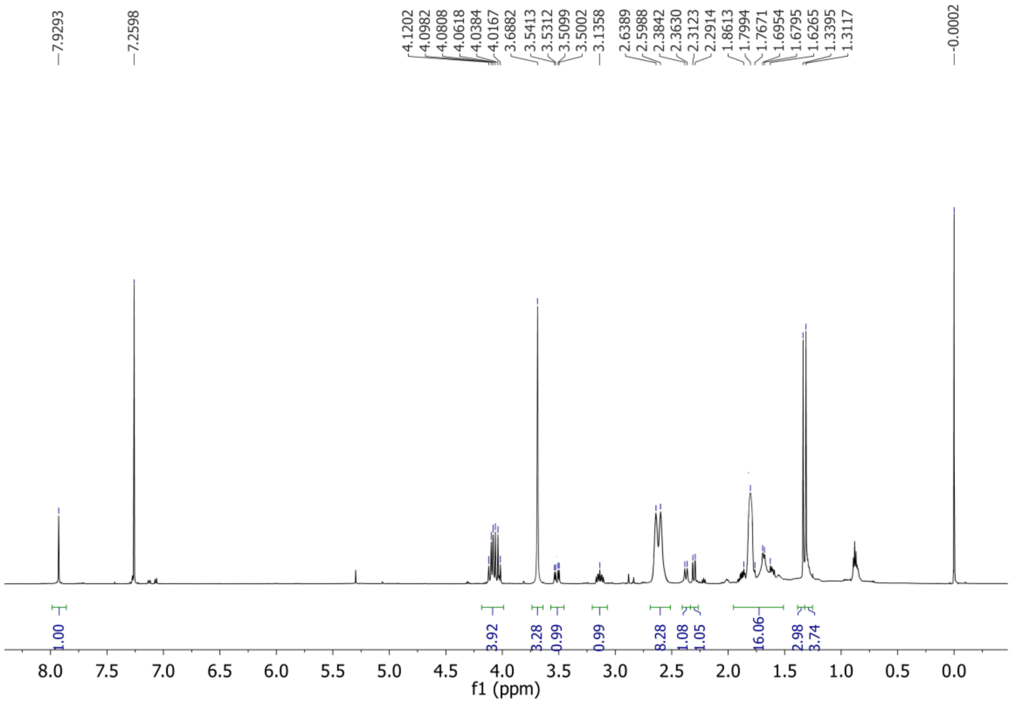

Figure S5. ^{1}H-NMR spectrum of compound **4e** (600 MHz, $CDCl_3$)

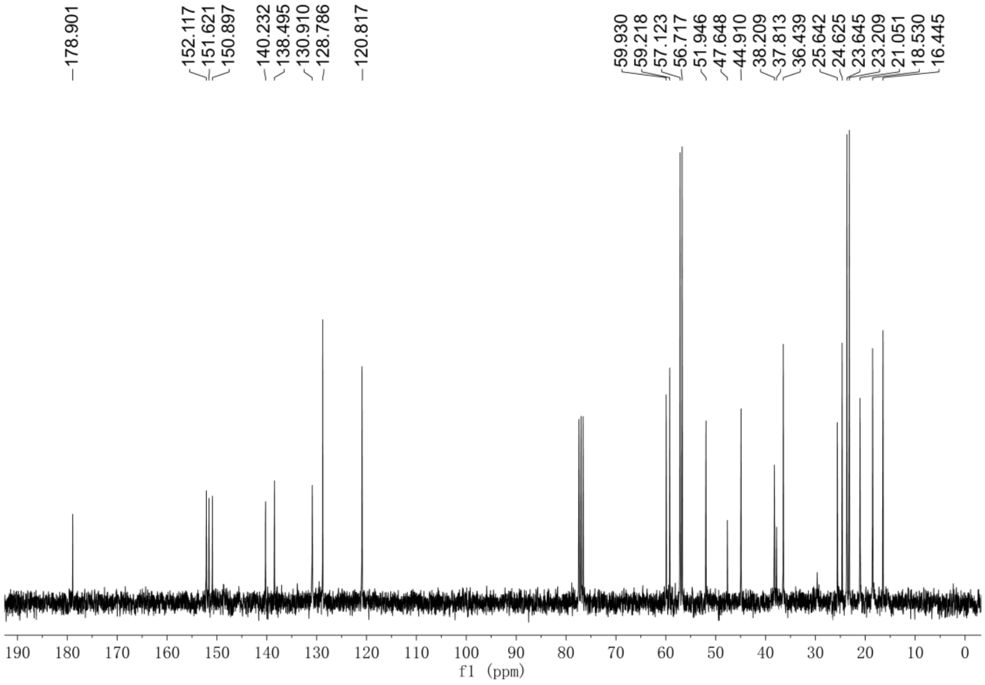

Figure S6. ^{13}C-NMR spectrum of compound **4e** (75 MHz, $CDCl_3$)

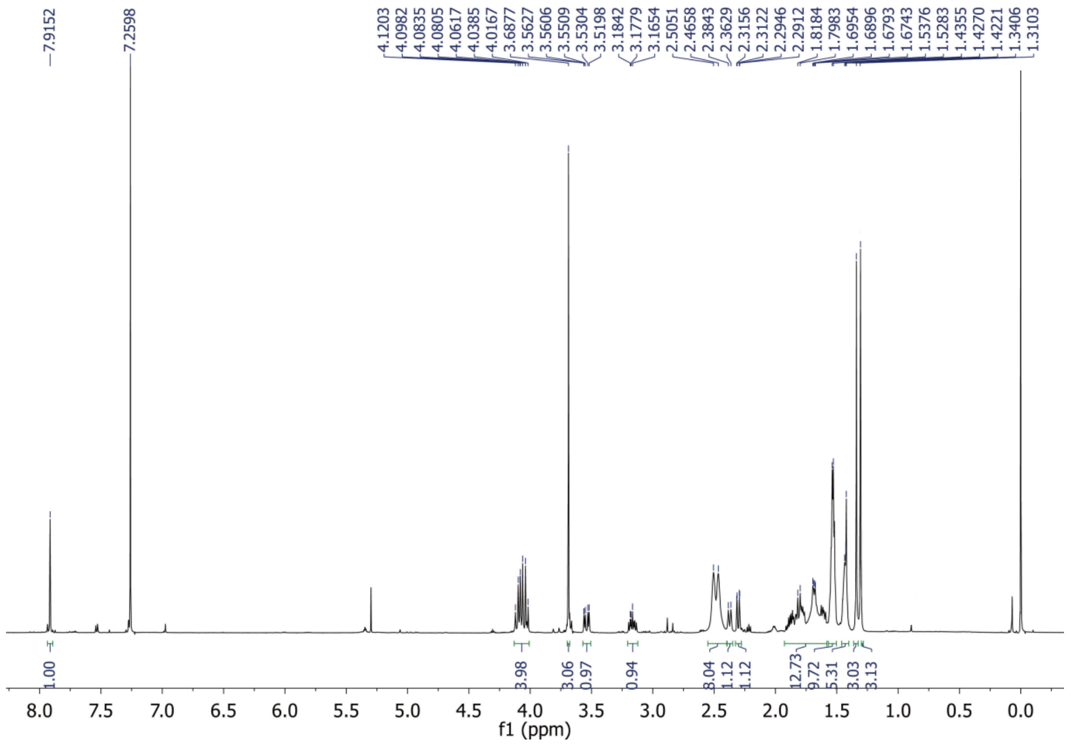

Figure S7. ^{1}H-NMR spectrum of compound **4f** (600 MHz, $CDCl_3$)

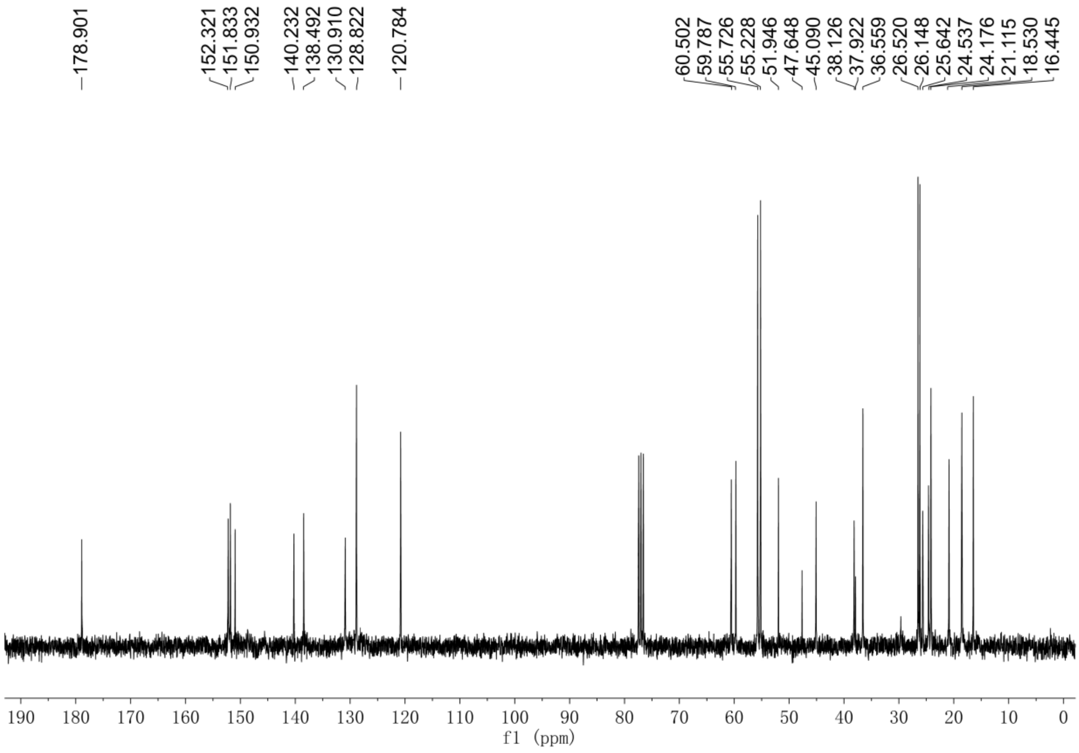

Figure S8. ^{13}C-NMR spectrum of compound **4f** (75 MHz, $CDCl_3$)

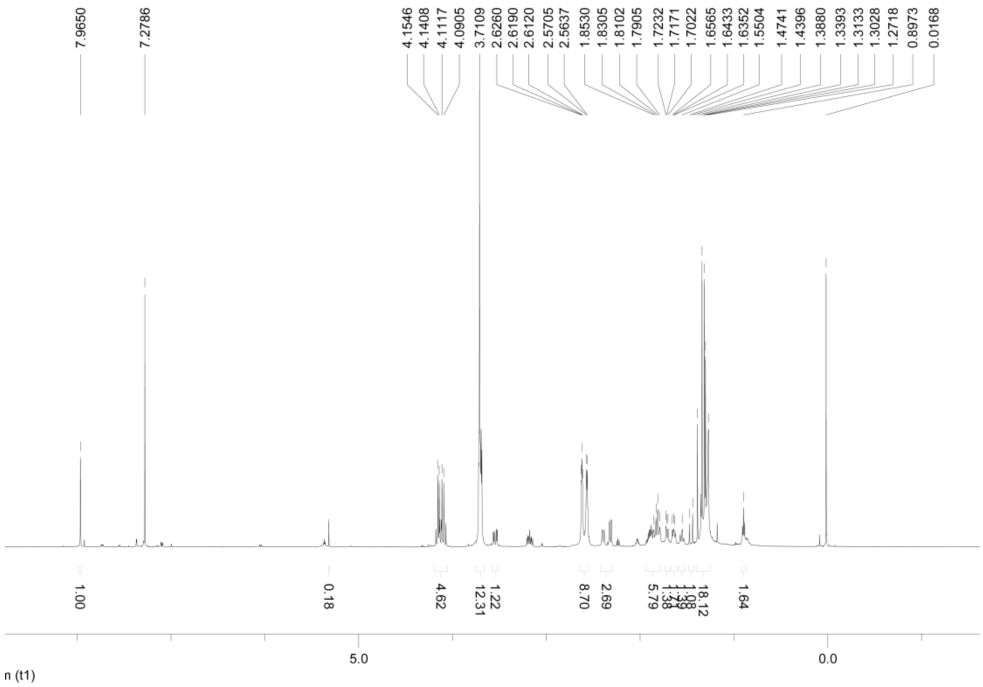

Figure S9. ^{1}H-NMR spectrum of compound **4h** (600 MHz, $CDCl_3$)

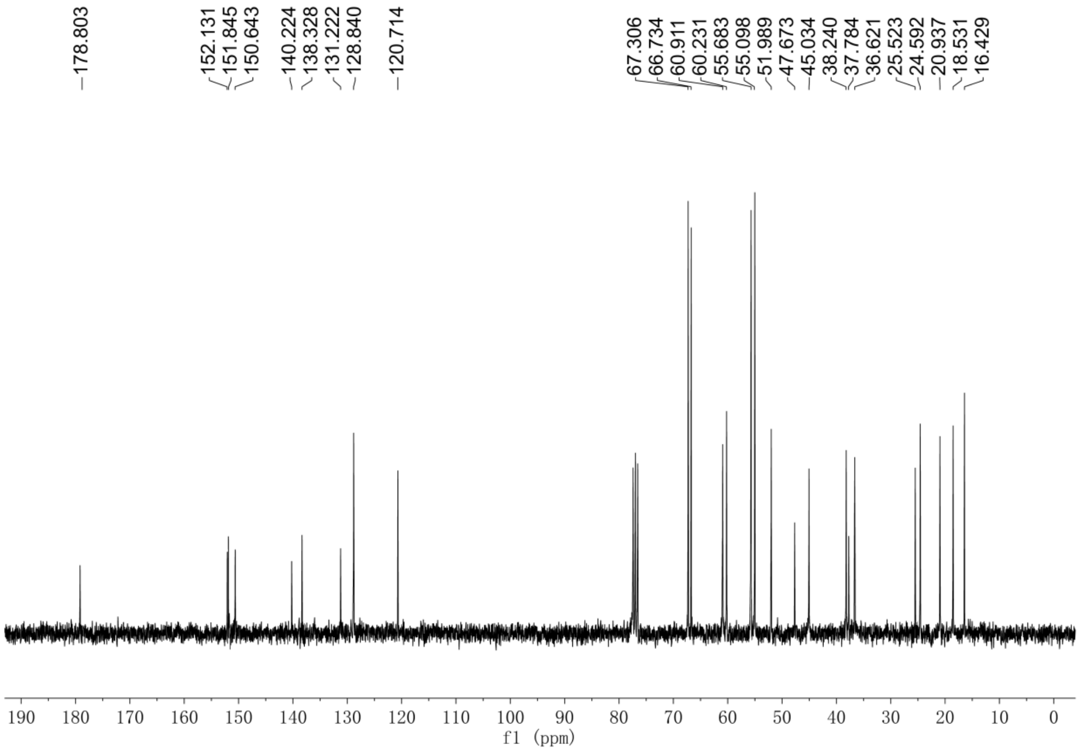

Figure S10. ^{13}C-NMR spectrum of compound **4h** (75 MHz, $CDCl_3$)

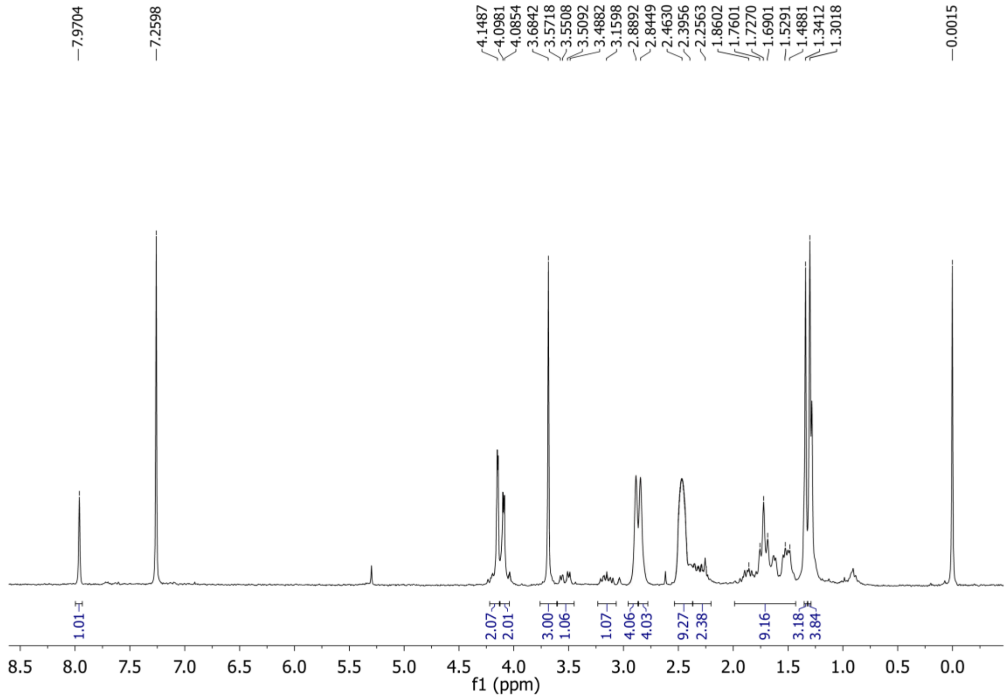

Figure S11. ^{1}H-NMR spectrum of compound **4i** (300 MHz, $CDCl_3$)

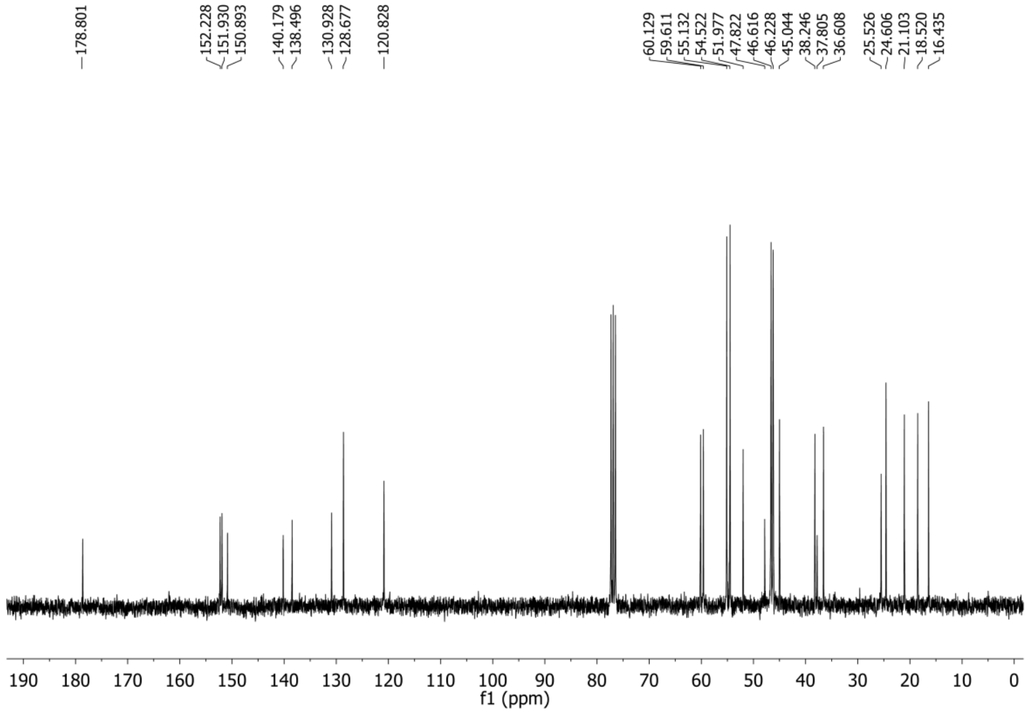

Figure S12. ^{13}C-NMR spectrum of compound **4i** (75 MHz, $CDCl_3$)

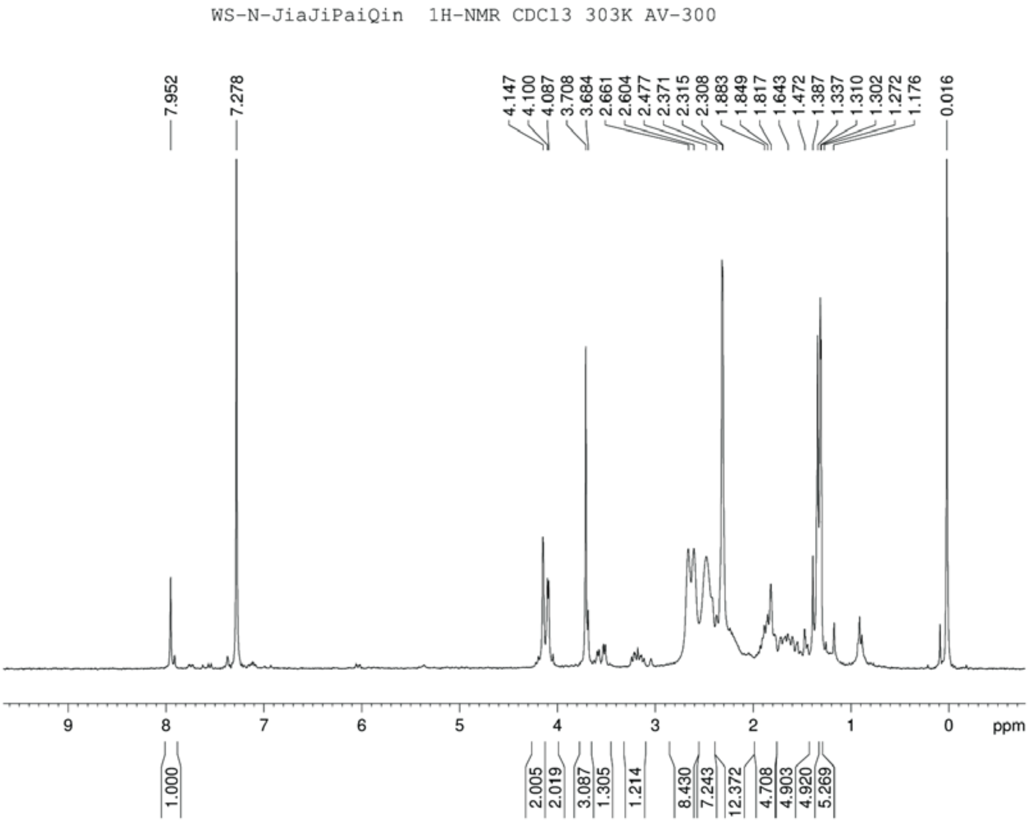

Figure S13. ^{1}H-NMR spectrum of compound **4j** (300 MHz, $CDCl_3$)

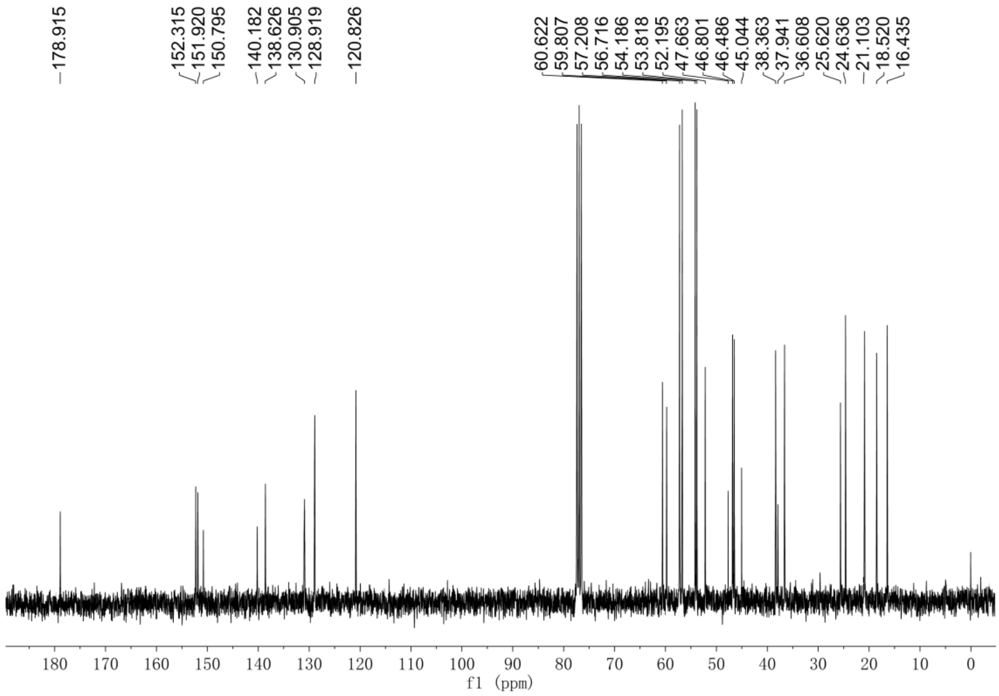

Figure S14. ^{13}C-NMR spectrum of compound **4j** (75 MHz, $CDCl_3$)

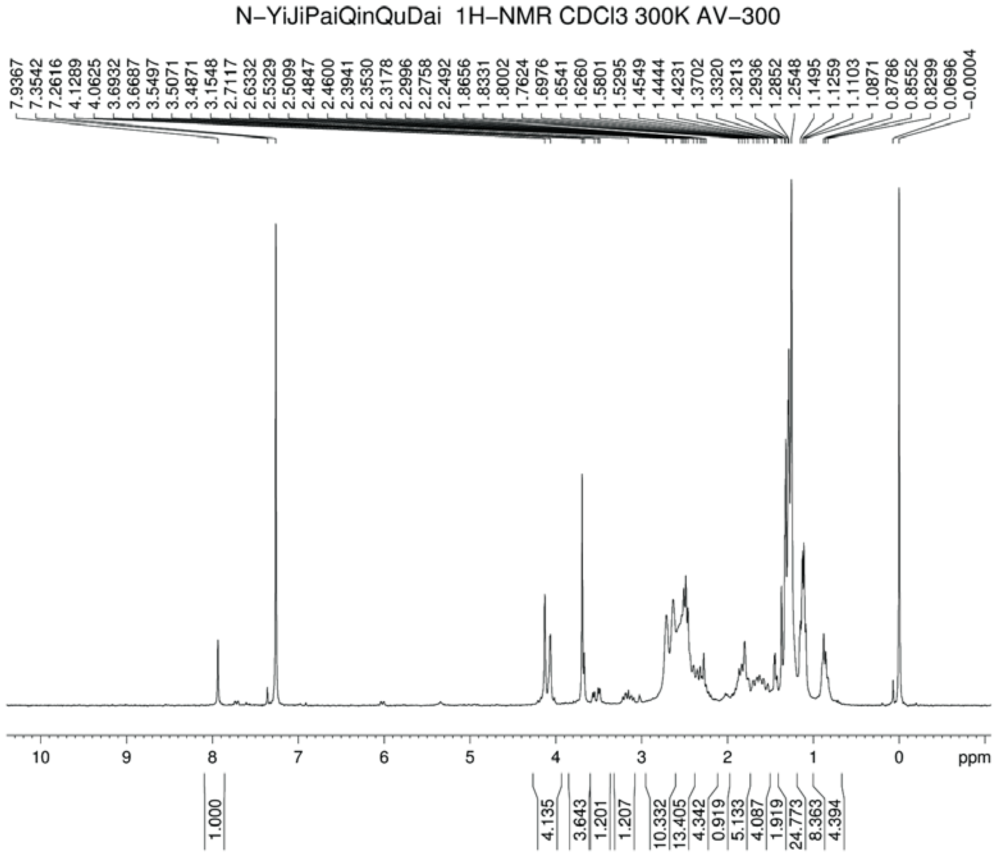

Figure S15. ^{1}H-NMR spectrum of compound **4k** (300 MHz, $CDCl_3$)

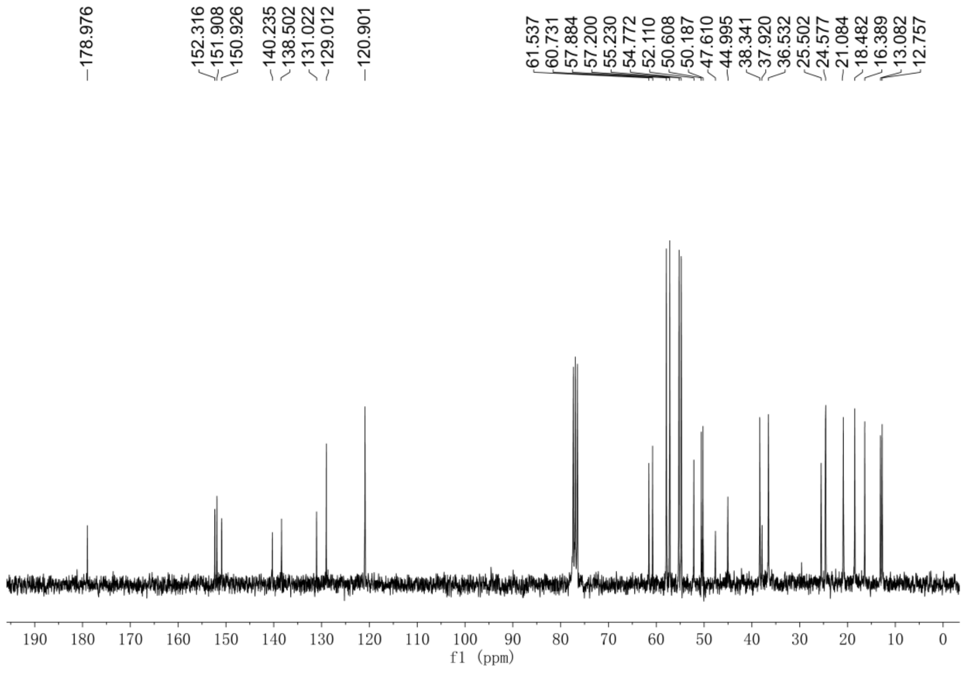

Figure S16. ^{13}C-NMR spectrum of compound **4k** (75 MHz, $CDCl_3$)

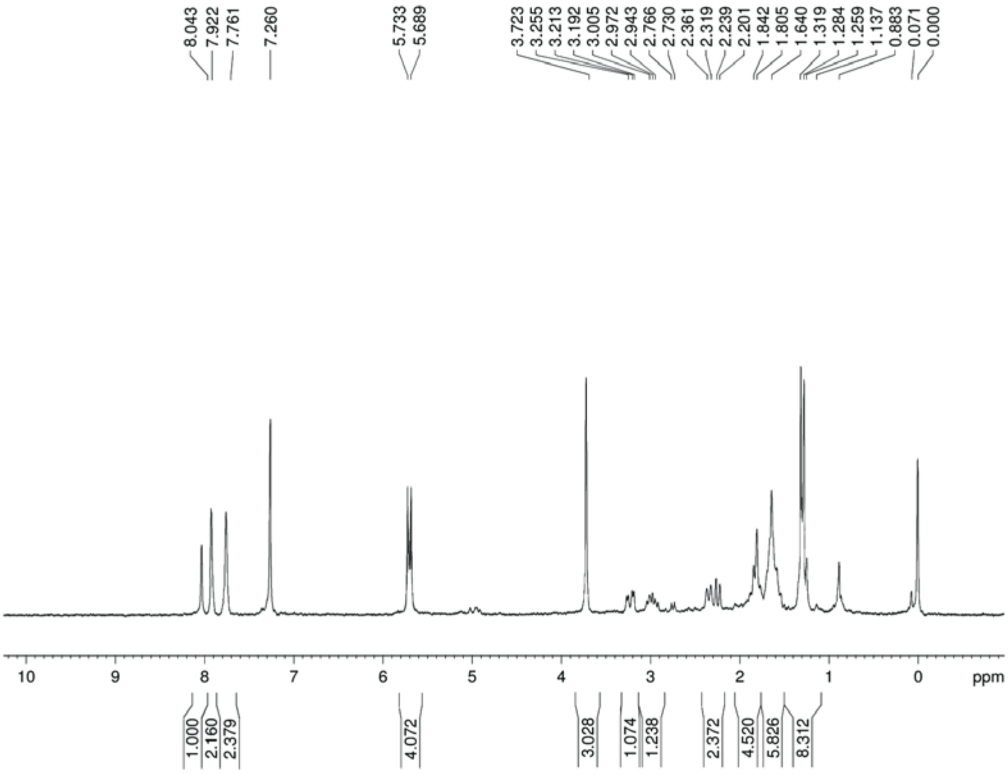

Figure S17. ^{1}H-NMR spectrum of compound **4m** (300 MHz, $CDCl_3$)

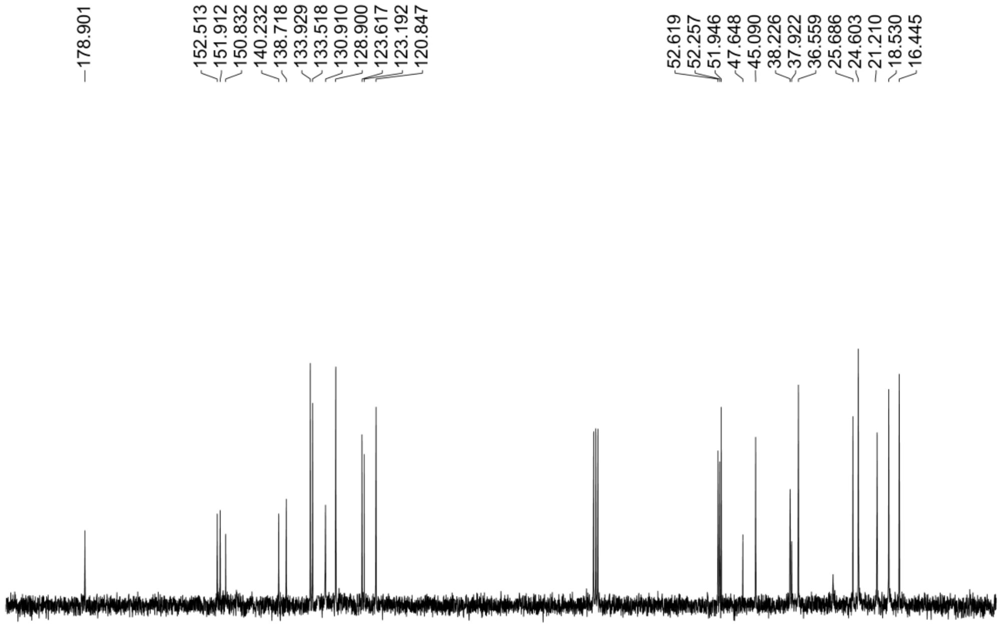

Figure S18. ^{13}C-NMR spectrum of compound **4m** (75 MHz, $CDCl_3$)

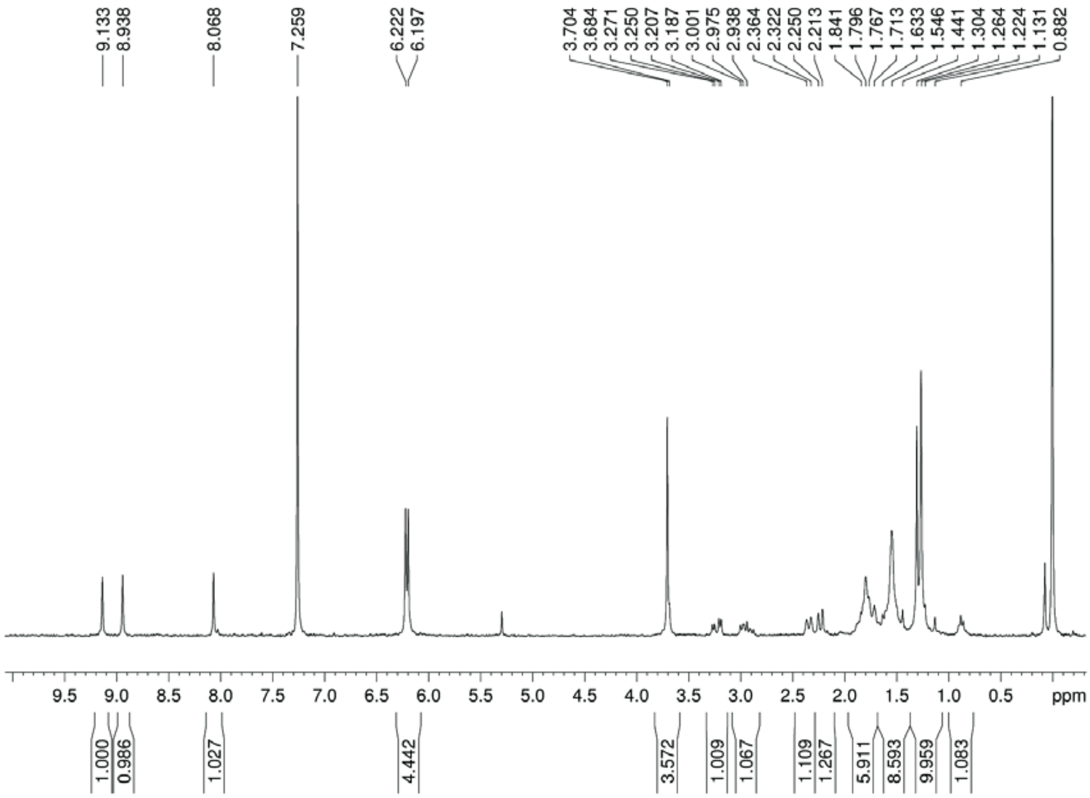

Figure S19. ^{1}H-NMR spectrum of compound **4n** (300 MHz, $CDCl_3$)

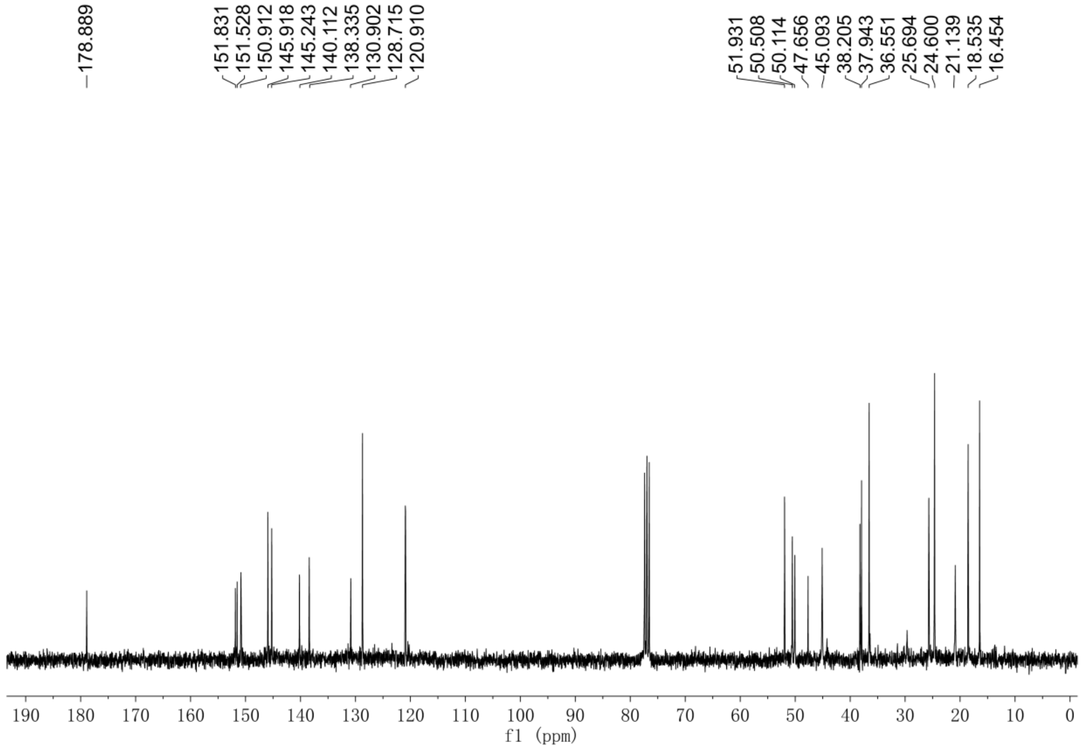

Figure S20. ^{13}C-NMR spectrum of compound **4n** (75 MHz, $CDCl_3$)

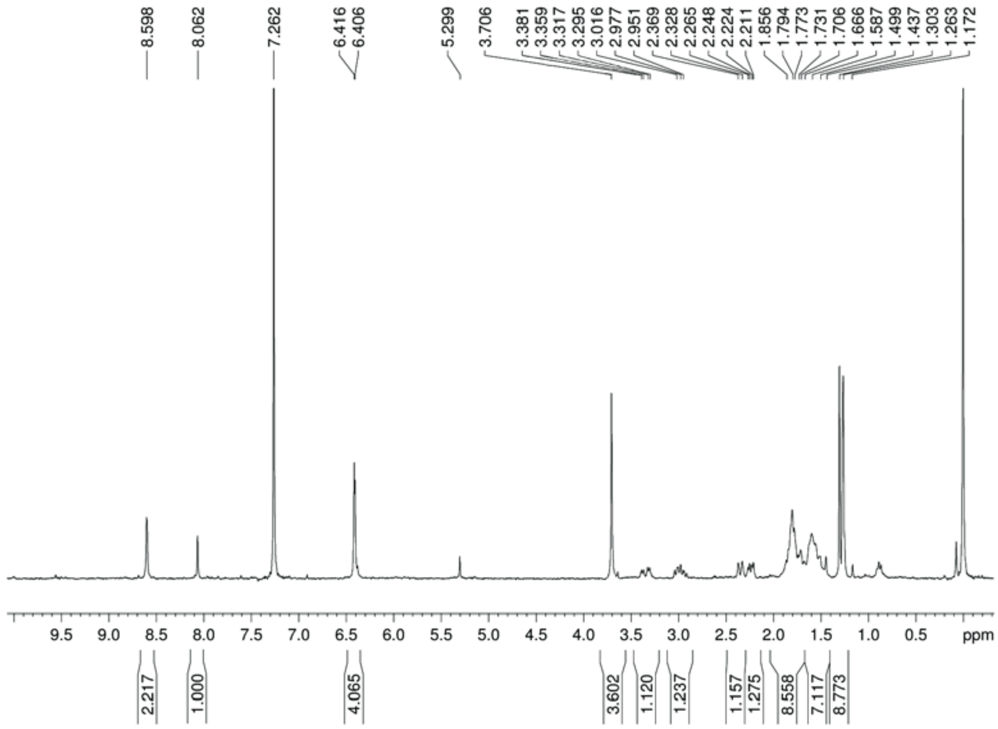

Figure S21. ^{1}H-NMR spectrum of compound **4o** (300 MHz, $CDCl_3$)

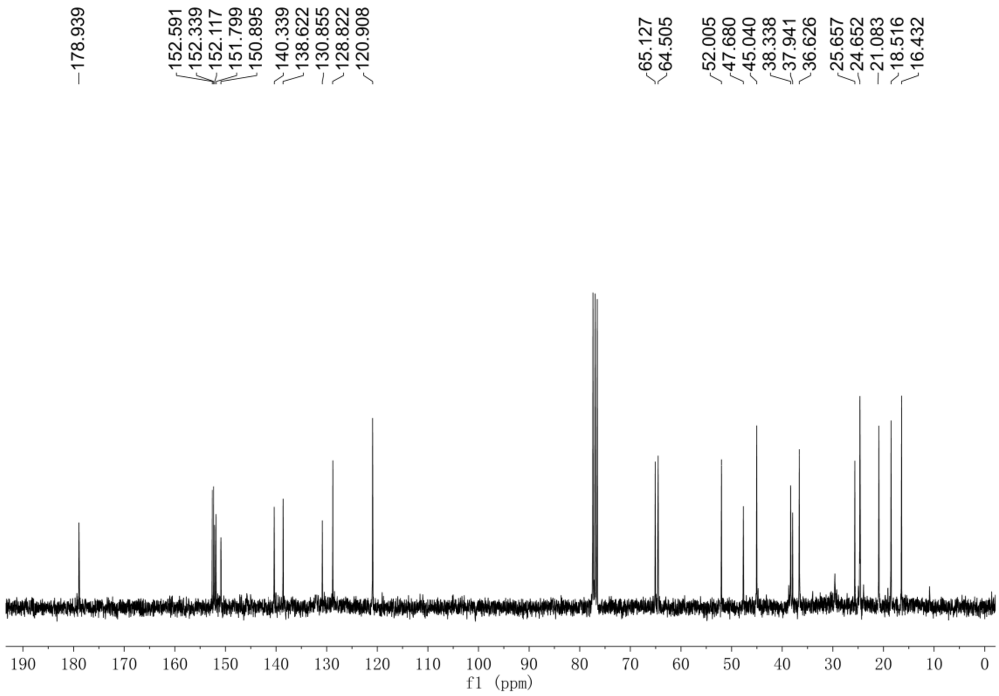

Figure S22. ^{13}C-NMR spectrum of compound **4o** (75 MHz, $CDCl_3$)
